# A Structure Based Study of Selective Inhibition of Factor IXa over Factor Xa

**DOI:** 10.3390/molecules26175372

**Published:** 2021-09-03

**Authors:** Sibsankar Kundu, Sangwook Wu

**Affiliations:** 1 Department of Physics, Pukyong National University, Busan 48513, Korea; sskundu@pharmcadd.com; 2 R&D Center of PharmCADD, Busan 48060, Korea; 3 Department of Chemistry, University of North Carolina, Chapel Hill, NC 27599, USA

**Keywords:** serine protease, blood coagulation factor, FIXa, FXa, target selectivity, structural alignment, sequence alignment, molecular docking, cross docking, pharmacophore modeling

## Abstract

Blood coagulation is an essential physiological process for hemostasis; however, abnormal coagulation can lead to various potentially fatal disorders, generally known as thromboembolic disorders, which are a major cause of mortality in the modern world. Recently, the FDA has approved several anticoagulant drugs for Factor Xa (FXa) which work via the common pathway of the coagulation cascade. A main side effect of these drugs is the potential risk for bleeding in patients. Coagulation Factor IXa (FIXa) has recently emerged as the strategic target to ease these risks as it selectively regulates the intrinsic pathway. These aforementioned coagulation factors are highly similar in structure, functional architecture, and inhibitor binding mode. Therefore, it remains a challenge to design a selective inhibitor which may affect only FIXa. With the availability of a number of X-ray co-crystal structures of these two coagulation factors as protein–ligand complexes, structural alignment, molecular docking, and pharmacophore modeling were employed to derive the relevant criteria for selective inhibition of FIXa over FXa. In this study, six ligands (three potent, two selective, and one inactive) were selected for FIXa inhibition and six potent ligands (four FDA approved drugs) were considered for FXa. The pharmacophore hypotheses provide the distribution patterns for the principal interactions that take place in the binding site. None of the pharmacophoric patterns of the FXa inhibitors matched with any of the patterns of FIXa inhibitors. Based on pharmacophore analysis, a selectivity of a ligand for FIXa over FXa may be defined quantitatively as a docking score of lower than −8.0 kcal/mol in the FIXa-grids and higher than −7.5 kcal/mol in the FXa-grids.

## 1. Introduction

While blood coagulation is an essential physiological process for hemostasis, abnormal blood coagulation has fatal impacts that lead to a number of coagulation-associated disorders. Arterial fibrillation causes clot formation which gives rise to a number of clinical (pathological) conditions, e.g., arterial and venous thrombosis, heart attack, ischemic stroke, pulmonary embolism, or cardiogenic stroke. All these conditions are, in general, categorized as thromboembolic disorders which continues to be one of the major causes of mortality and disability in the modern world. Anticoagulants are the established treatments to manage thromboembolic disorders [1,2,3].

Heparin and warfarin are the earliest therapies as anticoagulants for thromboembolic disorders, but these agents regulate the coagulation process through the indirect pathway and have restricted use due to their bleeding risk [4]. In recent years, several FXa (Factor Xa) inhibitors have been approved as the direct oral anticoagulants (DOAC) by the FDA for the treatment of these thromboembolic disorders, but they are not completely devoid of bleeding risk since this target falls under the common pathway [1,5] of coagulation process. Some of the FXa inhibitors are apixaban [6], rivaroxaban [7], letaxaban [8] and eribaxaban [9]. Even though these direct oral anticoagulants (DOAC) manage the risk of bleeding in a better way compared to warfarin and heparin, they are also not completely free from bleeding risk and hence these drugs should be prescribed with proper caution [10,11,12]. Therefore, the search for safer anticoagulants is still a challenging area of research.

To overcome the bleeding risk partially, an alternative therapeutic strategy was hypothesized and proposed towards the discovery of the therapeutic agents that can selectively regulate the intrinsic pathway without affecting the extrinsic pathway and the common pathway, and in turn manage the subtle balance of clot formation and the fluidity of blood. Inhibition of coagulation factors that reside on the intrinsic pathway has emerged as the attractive target for thromboembolic disorders with a potentially diminished bleeding risk. Selective inhibition of FIXa that falls under the intrinsic pathway, which is right above FXa in the downstream coagulation propagation (Figure S1, shown in the Supplementary Materials), is hypothesized and validated for improved bleeding related risk with similar efficacy compared to the FXa inhibitors. The basic tenet of this strategy is the selective regulation of the intrinsic pathway through selective therapeutic agents that would not be affecting the targets in the extrinsic pathway and the common pathway [1,2,3,10,11,12,13,14].

A number of molecular modeling and computer-aided design studies have been reported for both FXa [15,16,17] and FIXa [18,19] along with some structure based investigations [20]. As per the therapeutic strategy described above, i.e., selectively inhibiting the intrinsic pathway without any change in the extrinsic pathway and the common pathway, FIXa is considered as the strategic therapeutic target for this study. In recent years several FIXa inhibitors have been developed by researchers that have similar binding sites and binding modes with the FXa inhibitors (including recently approved FXa drugs). Due to this similarity in binding sites and binding modes, any inhibitor designed for FIXa would likely have an affinity toward FXa or vice versa. Hence, the selective inhibitor design for FIXa is very critical toward the management of the clinical condition. Many lead optimization programs and a number of experimental data also support the necessity of selective design FIXa inhibitors [10,11,12,13,14,21,22].

In this work, molecular modeling methods were employed to derive the criteria for binding of the ligands in the two coagulation factors that lead to selectivity. Structural alignment and analysis of the binding site were performed to understand the structural similarity and differences in the two coagulation factors and their impact on the binding of the ligand. Sequence alignment and analysis was also carried out to understand the changes in the binding site residues in these two coagulation factors. This analysis shows the conserved and non-conserved residues of the binding site, which play a critical role in the structural deformations of the ligand binding site and impact the ligand protein interaction that might be associated with the selectivity of binding. Cross docking experiments were performed to understand how the FIXa ligands fit in shape and size to the FXa crystal positions and vice versa. Pharmacophoric analysis were performed to determine the distribution pattern of the major interaction sites along the ligand (pharmacophore model) in these two sets of proteins to derive the criteria for selectivity. In summary, three major analyses were performed: (a) structural similarities and differences in these two sets of proteins and the role of conserved and non-conserved residues to the binding site shape, size and interaction that leads to selectivity; (b) how cross docking discriminates binding of ligands with the two coagulation factors; and (c) how pharmacophore modeling provides selectivity criteria for binding of the ligands.

## 2. Results and Discussion

### 2.1. Protein Structural Alignment

**Figure 1:**
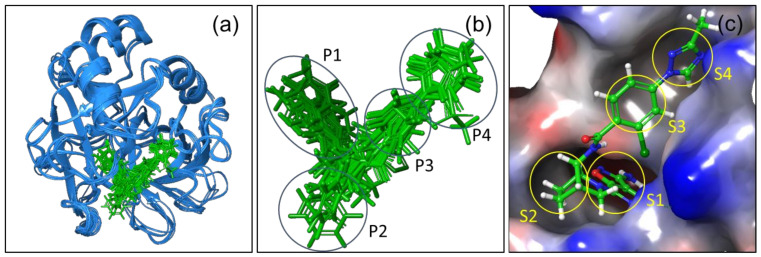
(**a**) The structural alignment of 12 co-crystallized structures of FIXa and FXa. (**b**) All the ligands in the co-crystallized structures are also aligned on same binding pocket with the same binding mode. (**c**) The binding mode of the co-crystallized ligand in the binding pocket with electrostatic potential surface representation with negative (red color), neutral (white color) and positive (blue color) patches on the surface. S1, S2, S3, S4 are the subsites of the long binding site and P1, P2, P3, P4 are the corresponding regions on the ligands that occupies those sites.

The structure based (SB) analysis of the two sets of proteins, FIXa (six structures) and FXa (six structures), from two coagulation factors, were performed by aligning all the co-crystal structures. The aligned structures are shown in Figure 1a. It was observed that the overall structural features, e.g., secondary structures, fold, and architecture of the tertiary structures are mostly aligned and conserved among these proteins, though there is a little misalignment in the loop regions, as shown in Figure 1a. All the aligned ligands are shown in Figure 1b (in green). It shows that the ligand binding modes are mostly conserved. All the ligands of FIXa and FXa are almost lying on the conserved binding site (Figure 1c) and in the same binding mode (i.e., the orientation of the similar groups and moieties of the ligands are aligned on each other), as shown in Figure 1b. The binding site and binding mode being identical in these two sets of proteins, it is interesting and at the same time challenging to study the selectivity between these two coagulation factors. The surface representation of the active site or the inhibitor binding pocket is shown in Figure 1c, where the S1 pocket is on the left-hand-side (LHS) and S4 pocket is on the right-hand-side (RHS). Figure 1c also shows the corresponding electrostatic potential around the active site of the protein, with negative (in red), neutral (in white) and positive (in blue) patches on it. The left-hand-side (LHS) of the ligand is buried deep inside the S1 pocket, shown in Figure 1c. The S3 and S4 pockets are on the right-hand-side (RHS) and crawl over the surface of the protein, shown in Figure 1c. The S2 pocket is occupied with the bent-region at the middle of the L-shaped molecule (Figure 1c and Figure S3).

**Figure 2:**
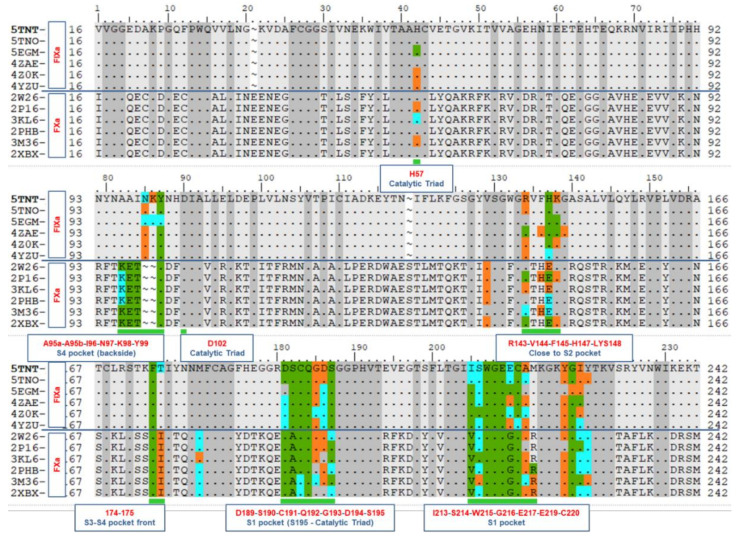
Human coagulation factor IXa and factor Xa sequence comparison were performed to identify the conserved residues within these two sets of proteins. There are 12 rows in this alignment representing 12 proteins. The first 6 are FIXa and next 6 are FXa. The dots represent the identical residues. The residues marked green indicates that they are within the distance of 3 Å from their corresponding ligands. Similarly, cyan represents residues within 4 Å and orange within 5 Å distance.

The ligand-protein proximity and their interactions were analyzed to understand the necessary and sufficient conditions of their contacts and interactions toward the selectivity of the ligands. All the heavy atoms and all the polar hydrogens of the proteins and ligands were considered during this contact analysis or proximity analysis. Three levels of distances were analyzed for each of the co-crystal structures, viz. 3 Å, 4 Å, and 5 Å, between ligand and protein residues. These residues are known as the binding site residues and are shown on the aligned sequences in Figure 2, in green within 3 Å, in cyan within 4 Å, and in orange within 5 Å.

**Figure 3:**
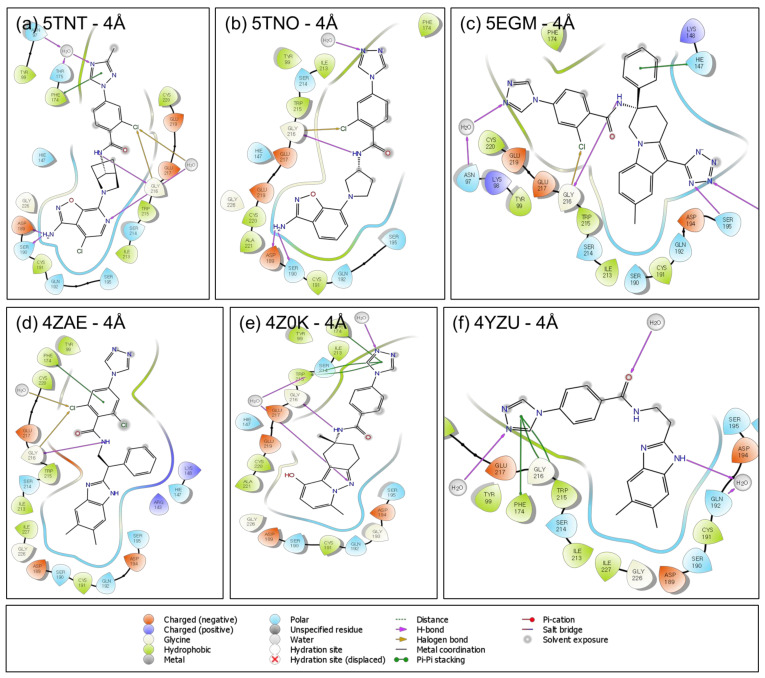
Protein–ligand interaction diagrams (LID) are shown here for all the FIXa co-crystal ligands within 4 Å distance: (**a**) for 5TNT, (**b**) for 5TNO, (**c**) for 5EGM, (**d**) for 4ZAE, (**e**) for 4Z0K and (**f**) for 4YZU.

**Figure 4:**
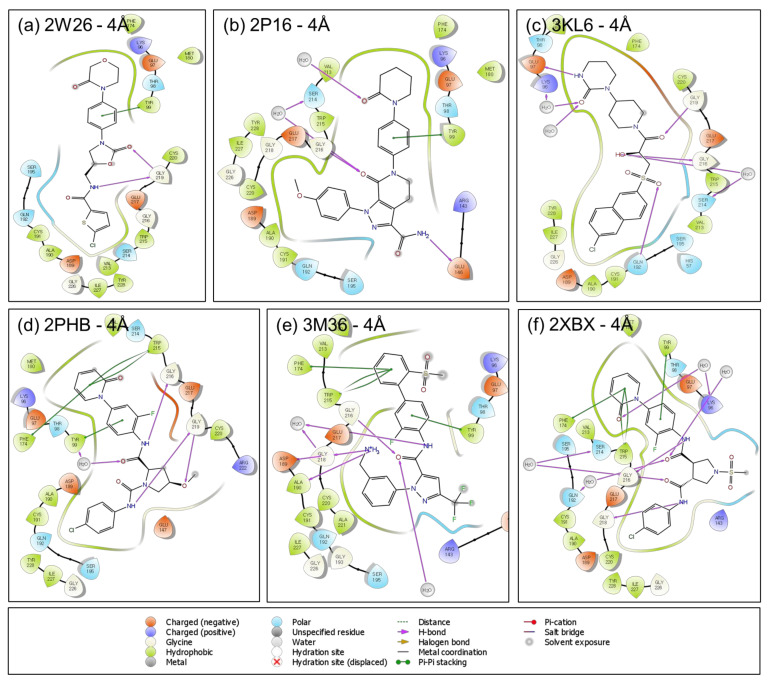
Protein–ligand interaction diagrams (LID) are shown here for all the FXa co-crystal ligands within 4 Å distance: (**a**) for 2W26, (**b**) for 2P16, (**c**) for 3KL6, (**d**) for 2PHB, (**e**) for 3M36 and (**f**) for 2XBX.

The protein–ligand interactions are also shown in the 2D ligand interaction diagram (LID) in Figure 3 and Figure 4 with 4 Å distance. Active sites of both the FIXa and FXa are very similar. The serine protease subfamily (S1A) of proteins has the long active site which is divided into several subsites or pockets designated as the S1, S2, S3, S4 based on the orientation of the bound inhibitor [23,24,25,26]. The shape and size of the S1 pocket, which is buried inside the proteins, is very similar in all the proteins selected in this study, but due to the differences in some of the residues in the two proteins, FIXa and FXa, there are very subtle differences observed in the S1 pocket that controls the binding of ligands to these targets (Figure S4).

### 2.2. Protein Sequence Alignment

The structural aspects of the binding pockets and their impact on binding affinity are discussed along with the aligned sequences to investigate the criteria for ligand binding and selectivity. Multiple Sequence Alignment (MSA) was performed followed by the structural alignment of these proteins (described in Section 3.1).

The interactions of the ligand with the protein for potency and selectivity were analyzed in detail from the co-crystallized structures of the ligands. The interactions that are required for the potency and selectivity of the ligands in FIXa and FXa are analyzed in this section. In 5TNT (FIXa target), the ABI (aminobenzisoxazole) ring (on the ligand) in the S1 pocket forms H-bonds with Asp189 and Ser190, and the NH of the amide group (on the ligand) forms another H-bond with the backbone of Gly216. A halogen bond is formed between the Cl atom in the phenyl ring and the backbone of the Gly216. Since the S3 and S4 pockets are surrounded by many aromatic rings, pi-staking interactions and aromatic H-bonds are predominant in this region. Some H-bonds are formed not directly with the residue but through the water molecules. One of them is formed here between the tri-azole ring and the residue Thr175 and Asn97. In 5TNO, all the interactions are similar to 5TNT except that pi-stacking interaction and indirect H-bond is formed with Asn97 through the water molecule. In 5EGM, the interactions are somewhat different. In the S1 pocket of 5EGM, an H-bond is formed between the ligand and the Ser195, His57 and Gly216. Another H-bond and a halogen bond (H-bond with halogen) are formed with the Gly216. An indirect H-bond is also formed with the Asn97 through the water molecule. In the case of 4ZAE, one aromatic H-bond is formed. The NH of amide (on the ligand) forms an H-bond with Gly216, and a halogen bond is also conserved with Gly216. Pi-pi-stacking interaction in the S4 pocket is formed and conserved. In the case of 4Z0K, S1 pocket H-bonds are present but no direct H-bond (through water) is formed with the residues. Ligand NH and Gly216 H-bond is present and pi-pi-stacking interactions are present and indirect H-bonds are formed in the S4 pocket. In the case of 4YZU, S1 pocket H-bonds are not present, Gly216 H-bond with ligand NH is also not formed, but Gly216 aromatic H-bond is formed, and pi-pi-stacking bonds are formed. These interactions are listed in Table 1.

On the other hand, for the FXa ligands, rivaroxaban (PDB ID: 2W26), in the S1 pocket ligand forms one normal H-bond and one aromatic H-bond with Gly219, but no H-bond in the deeper or buried part of the pocket. In the S2 pocket, the ligand makes one H-bond with the Gly219. In the S3 and S4 pockets, one pi-pi-stacking interaction and one aromatic H-bond is formed. In apixaban (PDB ID: 2P16), the S1 pocket forms one aromatic H-bond (H-bond with aromatic ring) with Gly218. In the S2 pocket amide NH (of the ligand) makes an H-bond with Gly146 and Cys191. In the S3 and S4 pockets, H-bond with Gly216 is formed (S3-S4 pocket), and a pi-pi-stacking interaction is formed by the ligand and some H-bonds through the water molecule. These interactions are further evaluated using pharmacophore modeling in Section 3.4. Though a set of interactions are necessary for potency and selectivity, it cannot provide a guidance on whether a newly designed molecule or a molecule from the database would be able to fit in the active site by shape and size. In order to evaluate these criteria, docking based methods were employed.

### 2.3. Molecular Docking

In order to perform a comparison of the effect of these small structural changes (structural deformation of the binding site) to the binding energy of the ligand, on a relative scale, various docking experiments were carried out with a representative set of co-crystallized ligand structures (listed in Table 2).

#### 2.3.1. Score in Place

Assuming the co-crystallized ligand poses as the experimental binding poses, the docking scores for these ligand-bound states were estimated the reference values of their binding energy. These values are listed in Table 2 and Table 3 for each co-crystallized ligand (indicated as “GScore” in kcal/mol). The inhibitor binding site of these two sets of proteins are usually very large. For the ease of analysis and understanding of the binding event, this long site was divided into four subsites or pockets, S1, S2, S3 and S4 [1,23,24,26]. Due to this binding pose, the fragment of the inhibitor that occupies the S1 pocket is surrounded by the protein from all sides and the fragments of the inhibitor that occupies the S3 and S4 pockets are crawling over the surface and one side of the ligand is exposed to solvent.

#### 2.3.2. Cognate Docking

Cognate docking is also known as self-docking or re-docking [27]. In this experiment, the co-crystallized ligands were extracted and separated as a single molecule. This molecule was then passed through the LigPrep and subsequently docked flexibly and freely without any constraint to its own crystal (i.e., its corresponding grid). In all the cases except one (PDB ID: 4ZAE), the docking poses were close to the co-crystal ligand with the lowest GScore and low RMSD values. The cognate docking poses are compared with the co-crystal ligands and are shown in Figure S5 (Supplementary Information). This experiment validates that the binding mode is unique in all these co-crystallized structures. In most of the cases, cognate docking provides a better score than the score-in-place. This might be due to better sampling of the conformations around the co-crystallized ligands. In one case (PDB ID: 4ZAE) the ligand was not docked to its crystallographic position. This might be due to worse sampling of the ligand during flexible docking. The structural stability of the protein–ligand system was also studied using molecular dynamic simulation and the details have been described in the Supplementary Information (Figures S6–S9).

#### 2.3.3. Cross Docking

When multiple co-crystallized structures of a protein are available with different bound ligands, docking of each bound ligand to the other co-crystal structures (or grids of the other co-crystallized structures) is generally described as cross docking [28,29,30,31,32]. The cross docking experiments were carried out to evaluate the selectivity of various binding sites. The binding site residues of the protein are rearranged differently and wrap around the different ligands to generate binding pockets of slightly different shapes and sizes (based on the chemical structure and the conformations of ligands). This is known as ligand induced deformation of the binding site (Figure S2). When the ligand is docked, it checks the complementarity on each grid point and summed it over all the grid point to generate the final score. When one ligand is replaced by another one, the complementarity score (docking score) is recalculated for new ligand. The protein grids are named by their corresponding “PDB-code-grid” (e.g., 5TNT-grid) and the co-crystallized ligands are named by the corresponding PDB-code-ligand (e.g., 5TNT-ligand) in this document. When nothing is mentioned, that indicates the co-crystallized structure (protein and ligand together).

The cross docking studies, performed on these two sets of proteins, show interesting results. There were 12 co-crystal ligands and 12 corresponding grids for docking (6 from FIXa and 6 from FXa). The cross docking results are summarized in Table 2 (for FIXa grids) and Table 3 (FXa grids). In each of these tables, two columns together represent the docking results of one protein grid and how they are ranked with their ascending scores (lowest energy is at the top). All the “GScore”s are shown in Table 2 for FIXa grids and in Table 3 for FXa grids. The cross docking was carried out with the “flexible” option in GLIDE SP docking which takes care of the extensive conformational sampling without any constraint. The most important fact is that all the FIXa ligands are self-docking to their own grids (except one in 4ZAE), but generate very bad “GScore”s with the FXa grids. This fact is in agreement with the small structural changes of FXa active site as discussed in the sequence-structure section. The potent and selective ligands of FIXa are not fitting properly with complementarity in the FXa grids during docking compared to the FXa potent and selective ligands. Moreover, since they are selective ligands of FIXa, the interactions are compatible with the FIXa binding site residues, and that is the reason it might not interact well with the FXa targets. Since 4ZAE is a potent and non-selective inhibitor, the 2XBX-ligand is giving a good docking score (top-ranked in 4ZAE grid) but 4ZAE ligand is docked to 2XBX grid with difficulties. Therefore, the compact binding pocket is encountered with many van der Waals clashes that play an important role to prevent binding. On the other hand, FIXa ligands do not score well in the FXa grids. Even though 4ZAE-ligand binds to the 2XBX-grid, the score is very low (−5.27) compared to the highest score in that grid (−14.83) (shown in Table 3). Several grids for these two sets of target proteins were taken (six for FIXa and six for FXa), as the shape of the active sites in all the FIXa proteins are not the same. They are slightly different in shape and size based on the chemical structure of the ligand, its conformation, and interaction. Interestingly, having this diversity in shape, none of the FIXa ligands are giving a reasonable score in FXa-grids. Conversely, all the FXa ligands are scored close to the top in the FIXa grids. In particular, 2XBX-ligand scored the top position in 4ZAE-grid. In most of the cases, FXa ligands are ranked second or third position from the top with a very high docking score with respect to the highest reference scores of the corresponding grid. On the other hand, in most of the cases, FIXa ligands are ranked at the bottom of the list on the FXa-grids and with very poor docking scores. Since there are a large number of co-crystallized structures available for FXa and a few are available for FIXa, this experiment can be used to check for selectivity while designing molecules for FIXa. Therefore, a new molecule that scores good (high) in the FIXa grids and scores bad (low) in the FXa grids is likely to be FIXa selective, but if it scores good (high) in FIXa grids and also scores good (high) in the FXa grids then it is difficult to decide its selectivity. To further confirm about their selectivity, distribution pattern of the major interaction sites were also evaluated in terms of pharmacophoric features.

### 2.4. Pharmacophore Modeling

Pharmacophore modeling provides information on major interaction centers of ligand molecules. All the ligands are from co-crystallized structures and their conformation is considered as the bioactive conformations. Therefore, the E-Pharmacophore model is used which provides the pharmacophoric features of the ligand that contribute the most to the binding energy. The pharmacophoric hypotheses have been developed by comparing the above pharmacophoric features.

A set of selective ligands were considered, to develop the pharmacophore based hypothesis. Three potent, two selective, and one inactive ligand were selected for FIXa and four FDA approved drugs and six potent ligands (Four FDA approved) were considered for FXa. In order to derive the pharmacophoric requirement for selectivity, the correlation between the pharmacophoric features and various pharmacological events were hypothesized, in a simplified manner. 

**Figure 5:**
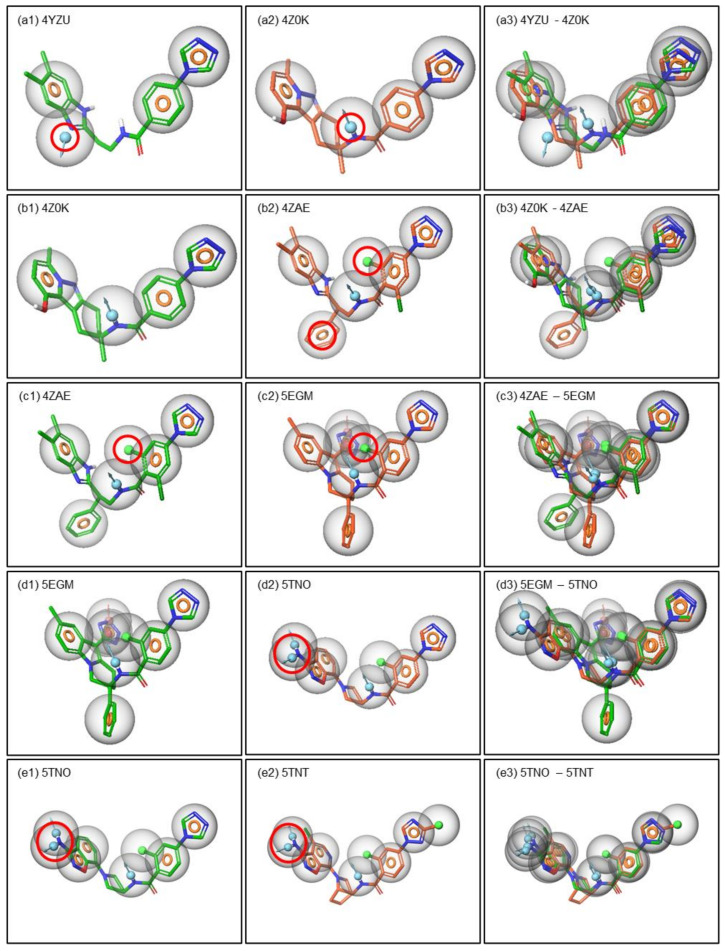
Pharmacophore based criteria is shown for potency and selectivity. The pharmacophoric descriptions are represented for potency (**a**), selectivity (**b**) and both together (**c**) for FIXa. Similarly, the pharmacophoric descriptions are represented for potency (**d**), selectivity (**e**) and both together for FXa. The pharmacophoric features are represented by colored spheres: H-bond donor in blue, H-bond acceptor in pink, hydrophobic in green and aromatic ring in orange ring.

In this context, the pharmacophoric features were compared and analyzed amongst these compounds to obtain the feature requirements for binding, activity, potency and selectivity within the FIXa co-crystallized molecules (Figure 5).

Amongst the FIXa compounds, 4YZU-ligand is the inactive one, and the compound 4Z0K-ligand is an active compound (IC_50_ values are 1000 nM or less). Pharmacophorically these two compounds are similar except the position of the donor (D) marked in RED circle in Figure 5(a1,a2). The position of the donor in the 4Z0K-ligand is forming an H-bond with the Gly216 which is also observed in the ligand interaction diagram (LID) of these two compounds (Figure 3). Therefore, the position of the donor (D) was hypothesized to be one of the basic criteria for biological activity. Though the amide group is present in the same position in 4YZU-ligand, the co-crystal structure shows that there is no H-bond formed between Gly216 and the ligand NH donor group. It is possible that the rotational flexibility of the middle part of this ligand fails to get sufficient exposure for the interaction. In a similar way, the biological activity of 4ZAE is improved by several folds compared to 4Z0K. The comparison of the pharmacophoric features in these two compounds showed that there are two additional pharmacophoric features in 4ZAE marked in RED circles in Figure 5(b2). One of them is aromatic (R) and the other one is hydrophobic (H) (this forms a halogen-H bond or halogen bond). These features are assumed to be responsible for the improvement of biological activity (i.e., potency) in 4ZAE. When the pharmacophoric features of 4ZAE are compared with that of 5EGM, where both of them are potent but non-selective, it is observed that the above hydrophobic (H) feature is also present in 5EGM, marked in RED circle in Figure 5(c2). Therefore, this hydrophobic (H) feature is hypothesized to improve the potency of these two compounds (4ZAE and 5EGM). Similarly, the potent non-selective compound 5EGM was compared with potent selective compounds 5TNO and 5TNT. An additional donor is hypothesized to give rise to selectivity to these compounds. These features are marked by RED circle and shown in Figure 5(d2,e2). The donor (D) interacts with the Asp189 and SER190 and forms H-bond in both the proteins which are also confirmed from the ligand interaction diagram (LID) in Figure 3.

**Figure 6:**
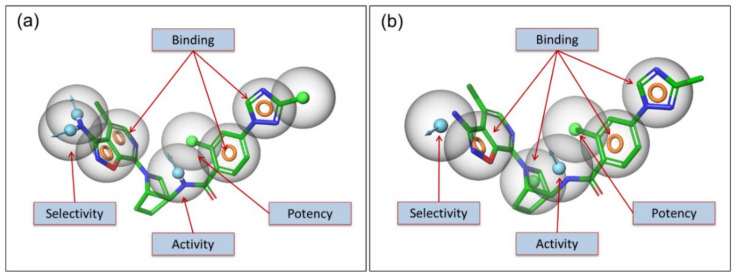
Pharmacophore based criteria is shown for potency and selectivity. The pharmacophoric descriptions are represented for potency, selectivity and both together for FIXa.

An additional donor (D) made it more selective as shown in Figure 6a. In an alternative method, the pharmacophore hypotheses was developed using pre-aligned co-crystal ligands in an automated way. This automated method provides an additional hydrophobic (H) feature (around the center of the molecule) to the hypothesis for binding as shown in Figure 6b. This model is also partially in agreement with the interactions listed in Table 1.

**Figure 7:**
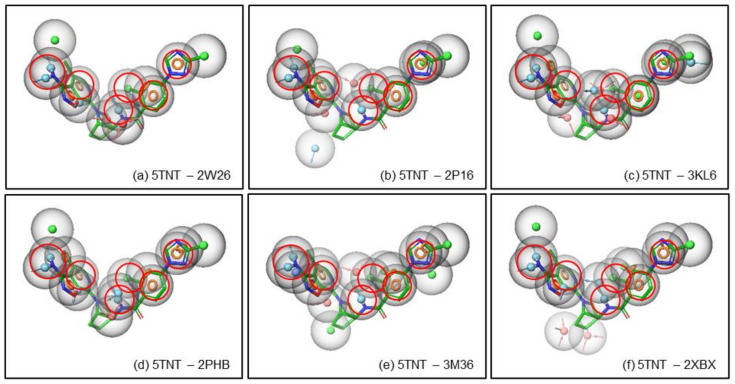
Pharmacophore based criteria is shown for potency and selectivity. The pharmacophoric descriptions are represented for potency (**a**), selectivity (**b**) and both together (**c**) for FIXa. Similarly, the pharmacophoric descriptions are represented for potency (**d**), selectivity (**e**) and both together (**f**) for FXa. The pharmacophoric features are represented by colored spheres: H-bond donor in blue, H-bond acceptor in pink, hydrophobic in green and aromatic ring in orange ring.

As the pharmacophore based potency and selectivity criteria were developed for the FIXa ligands, these criteria were compared with the FXa ligands to validate the above selectivity criteria. The pharmacophoric hypotheses of FLXa presented in Figure 6 are superposed with the pharmacophoric features of the FXa ligands in order to compare them. It is observed in Figure 7, that of the pharmacophoric features in all six FXa ligands, one or two pharmacophores are matching out of the minimum requirement of 4–7 pharmacophoric features for the potency and selectivity criteria for FIXa ligand. In fact, the activity and potency criteria of having a donor (D) that can form an H-bond with the Gly216 in FIXa is not present in most of the FXa ligands, and even when it is present it sometimes it does not form an H-bond to Gly216. The selectivity criteria developed for FIXa, i.e., the donor (D) that forms an H-bond with Asp189 and Ser190, is not matched with the FXa ligands, shown in Figure 7. This is important to note that Ser190 is present only in FIXa and not in FXa, therefore, this criterion is considered to be critical for selectivity.

## 3. Materials and Methods

### 3.1. Dataset

**Figure 8:**
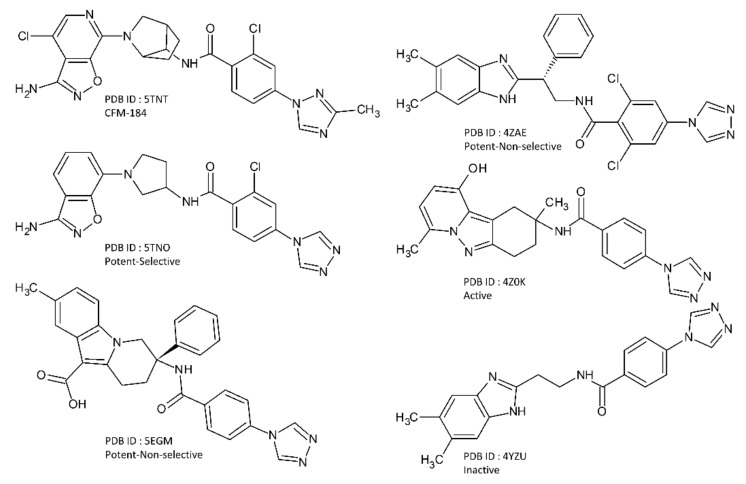
The 2D chemical structure of the co-crystallized FIXa ligands.

**Figure 9:**
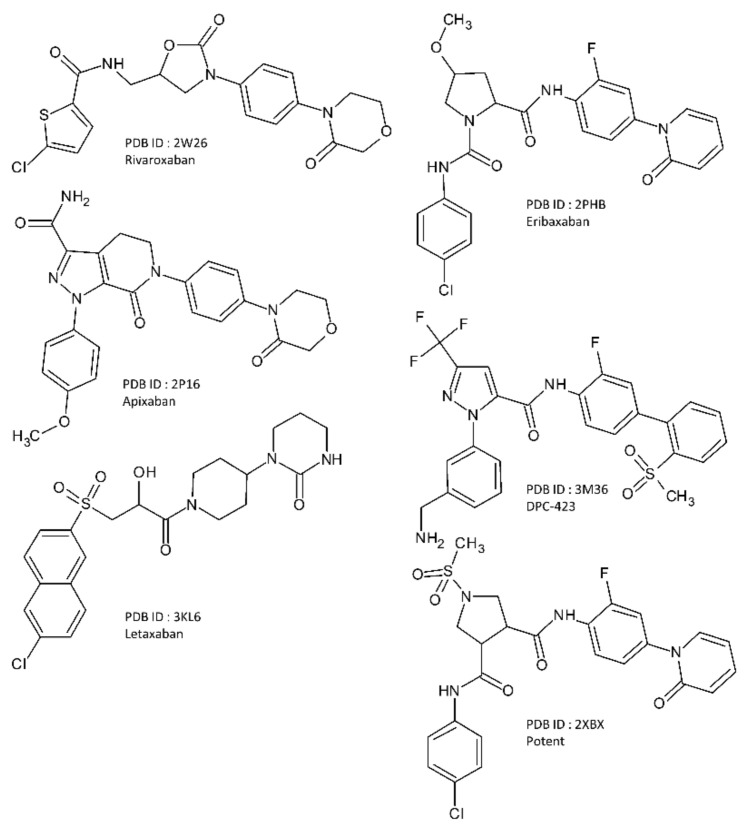
The 2D chemical structure of the co-crystallized FXa ligands.

A total of 12 co-crystal structures were selected for this work out of which 6 are from FIXa bound with 6 different ligands and 6 are from FXa bound with 6 different ligands. These structures were obtained from the RCSB Protein Data Bank (rcsb.org) [33,34]. The PDB ID of these structures are 5TNT [10], 5TNO [10], 5EGM [12], 4ZAE [11], 4Z0K [14], and 4YZU [14] for FIXa, and 2W26 [7], 2P16 [6], 3KL6 [8], 2PHB [9], 3M36 [35], and 2XBX [36] for FXa. They are listed in Table 4. The 2D chemical structures of the co-crystallized FIXa ligands are shown in Figure 8, and co-crystallized FXa ligands are shown in Figure 9.

With the availability of these high-resolution X-ray co-crystal structures of the protein ligand complexes of the two coagulation factors (FIXa and FXa) with diverse ligands, a systematic structure based analysis of these ligands was performed to understand the differential ligand binding affinity and selectivity. As the co-crystal ligands provide the experimental pose of binding and bioactive conformations, the accuracy of the model was expected to be higher in this structure based study. Some of the FXa inhibitors are FDA approved drugs (apixaban, rivaroxaban, letaxaban, eribaxaban) and some of them are optimized lead molecules. One Preclinical Candidate (PCC) of FIXa, CFM-184, was also included in this study. A few non-selective inhibitors of FIXa (5EGM, 4ZAE) and one inactive inhibitor (4YZU) were also included in this study. They are listed in Table 4. The compounds with IC_50_ values < 100 nM are considered as potent, IC_50_ values > 100 nM and <1000 nm are considered as active and IC_50_ values > 1000 nM are considered as inactive. The compounds are considered to be selective towards one target when the IC_50_ values of the other target is at least 500 time higher.

### 3.2. Protein Preparation

The proteins used in this study were prepared using the Protein Preparation Module of Schrodinger Software Suite, Schrodinger LLC, New York, NY, USA, 2020 [37,38] to make them suitable for this study. This process assigns the proper bond order to all the entities, generate hydrogens, generates correct ionization states of all the residues (using PROPKA at pH = 7.0), and determines the proper orientation of hydrogens by performing H-bond network optimization over the whole protein. The missing side chains and loops were built and modelled, wherever needed, using the PRIME module of the Schrodinger Suite of Programs. All the crystal waters beyond 5 Å of ligands were removed and the protein ligand system was finally minimized to prepare the protein to a realistic physiological state.

### 3.3. Protein Structural Alignment

“Protein Structure Alignment” panel in Schrodinger Software Suite, Schrodinger LLC was used to align the structures of all the proteins used in this study. All residues were considered for the alignment.

### 3.4. Protein Sequence Alignment

Sequence Alignment of all the proteins were performed using “Multiple Sequence Viewer” module of Schrodinger Software Suite, Schrodinger LLC.

### 3.5. Ligand Preparation

All the ligands used in this work were first prepared using the LigPrep module of Schrödinger Software Suite, Schrodinger LLC [37,38]. This process assigns the ionization state(s) of the ligands at the physiological condition (pH = 7.4) and generates 3D conformations of the possible tautomeric states and stereoisomers of the ligands. The prepared ligand was used for various calculations within the Schrödinger Software Suite, Schrodinger LLC.

### 3.6. Molecular Docking

Standard precision (SP) Molecular docking was performed using GLIDE module (Grid-based Ligand Docking with Energetics) [37,38] within the Schrödinger Software Suite, Schrodinger LLC. Since the co-crystallized structure of the ligand is considered to be the best experimental binding pose, the binding energy of the co-crystallized ligand was estimated for the bound state by calculating the score in this bound state using the “score-in-place” option in GLIDE. This score represents the binding energy and is considered to be the reference score for the corresponding protein–ligand system. The GLIDE docking score is represented as GScore, and it provides an estimate of binding energy in kcal/mol. This reference GScore was calculated for all the co-crystallized structures used in this study. The cognate docking or self-docking or re-docking was performed using an unbiased (without any constraint) SP docking protocol with a conformational sampling of the ligands (flexible docking option in the GLIDE). The same unbiased flexible SP docking protocol was used to perform the cross docking experiments in this work. In this experiment, the grid was generated from each ligand bound co-crystal structure. The ligands were separated from these 12 co-crystal structures, and prepared using LigPrep and docked to the 12 prepared grids. The docking results are analyzed in the following section.

### 3.7. Pharmacophore Modeling

Pharmacophore hypotheses were developed using the E-Pharmacophore model in PHASE (Pharmacophore Alignment and Scoring Engine) [39,40] within the Schrödinger Software Suite, Schrodinger LLC. The E-Pharmacophore model generates hypotheses from the co-crystal structure of protein ligand complexes based on complementarity between protein and ligand either automatically or manually. The automatic generation uses the GLIDE XP scoring terms to determine the most important features (interactions) contributing towards binding. In the manual options, the features can be picked or selected manually. Once they are generated, the pharmacophoric features and their positions in space are manually compared to generate a set of pharmacophoric criteria for their binding, activity, potency and selectivity. Six types of pharmacophoric features were generated in this study. These pharmacophoric features are represented either by colored spheres or by the colored ring in 3D. H-bond donors (D) are represented by light blue spheres, H-bond acceptors (A) by pink spheres, positives (P) charges by dark blue spheres, negatives (N) charges by dark red spheres, hydrophobic (H) groups by green spheres and aromatic (R) groups by orange rings. A combination of these features that correlates with the biological activity or potency is defined as pharmacophore hypotheses.

## 4. Conclusions

Through the structural analysis and sequence analysis, the binding site deformations (small structural changes around the binding site) were shown to be very small. These small structural changes could not shed much light on the selectivity, rather it describes the objective of this work and provides motivation for further investigation toward cross docking and pharmacophore modeling. With the cross docking experiments, an overall shape and size complementarity was estimated by the relative scores. Though the overall scores were sometimes good (e.g., 2XBX-ligand of FXa scored well in 4ZAE-grid of FIXa), these experiments still cannot resolve the selectivity issues, since the distribution pattern of the various important interaction sites along the active site are different. The pharmacophoric analysis was performed using the E-Pharmacophore method. This provides more insight into selectivity within these two coagulation factors. None of the FXa inhibitors matched with any of the patterns of pharmacophoric pattern with the FIXa inhibitors. For a ligand to be selective for FIXa against FXa (for the particular binding mode) it must be: (a) top scoring (<−8.0 kcal/mol) in the FIXa-grids (as per Table 2), (b) low scoring (>−7.5 kcal/mol) in the FXa-grids (as per Table 3), and (c) matching pharmacophoric pattern (as per Figure 6).

We predicted ADMET (Absorption, Distribution, Metabolism, Excretion, and Toxicity) descriptors for twelve ligands using the QikProp module. We observed that three ligands being considered for FIXa inhibition (5TNT-ligand, 5EGM-ligand, and 4ZAE-ligand) exhibited aqueous solubility values which were out of range (QPloS and CIQPlogS) (Table S3). QPlogS and CIQPlogS describe predicted aqueous solubility and conformation-independent predicted aqueous solubility for small molecules, respectively. One ligand being evaluated for FXa (2XBX-ligand) exhibited an aqueous solubility value that is considered out of range in QPlogS. The predicted aqueous solubility values for 5TNT-ligand and 2XBX-ligand were estimated to be −6.79 and −6.58, respectively. These values were just outside of the allowable range in QPlogS (−6.5~−0.5). Two lead compounds being evaluated for FIXa (5EGM-ligand, 4ZAE-ligand) exhibited aqueous solubility values which were significantly out of range. These findings suggest that the selected drug candidates should be further optimized by improving their aqueous solubility. ADMET descriptors for the twelve ligands are listed in Table S3.

This study aimed to develop a framework for the analysis of selectivity from a pharmacodynamics point of view and is a very critical initial step toward a drug design process. Systematic analysis of the binding modes of different classes of compounds and their interactions with the protein was performed, which is consistent with the previous works [1,18,41]. High similarity in the active sites of these two targets poses a challenge to the discovery of selective ligands for these targets. Since a large number of co-crystallized structures are available for the various lead compounds of FIXa and FXa, their biological activity data are also available in the literature. A more detailed analysis can be carried out with a bigger data set to refine the criteria for selectivity further.

## Figures and Tables

**Table 1:** The list of interactions in the two sets of proteins.

PDB ID		H-Bond							Halogen Bond	Pi-Pi Stacking		
5TNT	FIXa	Asp189	Ser190	Gly216					Gly216	Phe174	-	-
5TNO		Asp189	Ser190	Gly216					Gly216	-	-	-
5EGM		-	-	Gly216					Gly216	-	-	His147
4ZAE		-	-	Gly216					Gly216	Phe174	-	-
4Z0K		-	-	Gly216					-	Phe174	Trp215	-
4YZU		-	-	-					-	Phe174	Trp215	-
2W26	FXa			-	Gly219				-		-	Tyr99
2P16				Gly216	-	Glu146			-		-	Tyr99
3KL6				Gly216	Gly219		Gln192	Glu97	-		-	Tyr99
2PHB				Gly216	Gly219				-	Phe174	Trp215	Tyr99
3M36		Asp189	Ala190	Gly216					-	Phe174	Trp215	Tyr99
2XBX		-	-	Gly216	Gly218				-	Phe174	Trp215	Tyr99

**Table 2:** Cross docking data with FIXa grids. Green cells with green text represent the FIXa ligands and the red texts indicate moderate binding energy of the corresponding ligand. A longer form of this Table is provided in the Supplementary Information (Table S1).

FIXa												
	Grid 5TNT		Grid 5TNO		Grid 5EGM		Grid 4ZAE		Grid 4Z0K		Grid 4YZU	
		Gscore (kcal/mol)		Gscore (kcal/mol)		GScore (kcal/mol)		GScore (kcal/mol)		GScore (kcal/mol)		GScore (kcal/mol)
1	5TNT	−9.95	5TNT	−9.65	5EGM	−9.85	2XBX	−9.86	4Z0K	−10.33	4YZU	−9.71
2	5TNT	−9.77	5TNO	−9.38	5EGM	−9.79	3KL6	−9.56	4Z0K	−9.50	4YZU	−9.63
3	5TNT	−9.60	5TNT	−9.18	5EGM	−9.17	2XBX	−9.41	5TNT	−8.81	3KL6	−9.49
4	2XBX	−8.29	5TNO	−8.89	4Z0K	−8.45	3M36	−9.35	5TNO	−8.81	4ZAE	−9.29
5	3KL6	−8.17	5TNO	−8.58	2XBX	−8.15	2XBX	−9.21	2XBX	−8.54	3KL6	−9.23
6	2XBX	−8.06	4Z0K	−8.58	2XBX	−8.12	2XBX	−9.15	5TNO	−8.52	2XBX	−9.09
7	2XBX	−7.89	2XBX	−8.54	2XBX	−8.10	2XBX	−9.06	5TNO	−8.50	3KL6	−9.03
8	2PHB	−7.81	4Z0K	−8.28	4Z0K	−8.04	2PHB	−8.97	4YZU	−8.36	3KL6	−8.98
9	2PHB	−7.70	2W26	−8.07	2XBX	−8.01	2XBX	−8.90	5TNO	−8.30	4YZU	−8.96
10	2XBX	−7.68	3KL6	−7.95	2XBX	−7.88	3M36	−8.89	5TNO	−8.20	4ZAE	−8.96
11	2XBX	−7.63	5TNT	−7.84	2XBX	−7.86	2XBX	−8.80	2XBX	−7.73	4YZU	−8.85
12	2XBX	−7.60	2PHB	−7.83	2W26	−7.85	3KL6	−8.72	4YZU	−7.73	3KL6	−8.81
13	2PHB	−7.57	2PHB	−7.82	2PHB	−7.60	2PHB	−8.71	2XBX	−7.68	2XBX	-8.73
14	2PHB	−7.53	2XBX	−7.78	2PHB	-7.49	2XBX	−8.71	2XBX	−7.66	2PHB	−8.70
15	2XBX	−7.47	2PHB	−7.68	2PHB	−7.47	2XBX	−8.66	2XBX	−7.61	2XBX	−8.62
16	2XBX	−7.47	3KL6	−7.68	2W26	−7.45	3KL6	−8.65	2XBX	−7.56	2XBX	−8.48
17	2XBX	−7.37	2PHB	−7.68	2PHB	−7.35	3M36	−8.62	5TNO	−7.52	2XBX	−8.48
18	2PHB	−7.36	2XBX	−7.65	2W26	−7.11	3M36	−8.62	2W26	−7.37	3KL6	−8.47
19	2PHB	−7.35	2W26	−7.62	2PHB	−7.05	5TNT	−8.61	2PHB	−7.35	2W26	−8.45
20	3KL6	−7.34	2XBX	−7.60	2PHB	−7.03	3KL6	−8.57	2XBX	−7.32	3KL6	−8.39
21	2XBX	−7.33	2PHB	−7.58	2PHB	−6.99	2XBX	−8.54	2XBX	−7.24	2XBX	−8.39
22	3KL6	−7.30	2PHB	−7.54	2W26	−6.98	2PHB	−8.49	2XBX	−7.19	3KL6	−8.39
23	3KL6	−7.21	2XBX	−7.46	2XBX	−6.98	2PHB	−8.46	3KL6	−7.18	2W26	−8.37
24	2PHB	−7.20	2PHB	−7.41	2W26	−6.97	2P16	−8.43	2W26	−7.13	2W26	−8.30
25	3KL6	−7.13	3KL6	−7.37	2PHB	−6.93	2PHB	−8.43	3KL6	−7.13	2XBX	−8.29
26	2PHB	−7.07	3KL6	−7.33	5TNT	−6.87	5TNT	−8.40	3KL6	−7.06	3KL6	−8.29
27	2XBX	−7.04	2XBX	−7.33	2PHB	−6.72	2PHB	−8.40	2PHB	−7.00	4ZAE	−8.19
28	2XBX	−7.04	2PHB	−7.31	2PHB	−6.63	2XBX	−8.40	2W26	−6.99	2XBX	−8.16
29	2PHB	−6.85	2PHB	−7.31	2PHB	−6.59	3KL6	−8.39	3KL6	−6.97	2XBX	−8.00
30	3KL6	−6.83	2XBX	−7.27	2W26	−6.45	3KL6	−8.31	2PHB	−6.95	2XBX	−7.93

**Table 3:** Cross docking data with FXa grids. Green cells with green text represent the FIXa ligands and the red texts indicate moderate binding energy of the corresponding ligand. A longer form of this Table is provided in the Supplementary Information (Table S2).

FXa												
	Grid 2W26		Grid 2P16		Grid 3KL6		Grid 2PHB		Grid 3M36		Grid 2BXB	
		GScore (kcal/mol)		GScore (kcal/mol)		GScore (kcal/mol)		GScore (kcal/mol)		GScore (kcal/mol)		GScore (kcal/mol)
1	3M36	−11.46	2P16	−11.48	2XBX	−12.95	2XBX	−11.90	3M36	−13.78	2XBX	−14.83
2	2XBX	−11.00	2XBX	−11.07	2XBX	−12.61	2XBX	−11.87	3M36	−13.73	2XBX	−14.81
3	2PHB	−10.91	2XBX	−11.03	2XBX	−12.56	3M36	−11.85	3M36	−12.41	2XBX	−14.58
4	2XBX	−10.89	2XBX	−11.02	2XBX	−12.55	3M36	−11.69	3M36	−12.34	2PHB	−14.26
5	3M36	−10.85	2XBX	−11.01	3KL6	−12.35	2XBX	−11.64	3M36	−11.65	2PHB	−14.09
6	2XBX	−10.80	3M36	−10.73	2PHB	−12.28	2XBX	−11.52	3M36	−11.35	2PHB	−13.00
7	2PHB	−10.73	3M36	−10.65	2XBX	−12.27	3M36	−11.49	2P16	−9.06	2PHB	−12.86
8	3M36	−10.72	2P16	−10.63	3KL6	−12.14	2PHB	−11.18	2XBX	−9.00	3M36	−12.68
9	2XBX	−10.47	2PHB	−10.47	2P16	−12.11	2PHB	−11.08	2XBX	−8.75	2XBX	−11.65
10	2P16	−10.34	2PHB	−10.42	2PHB	−11.96	2PHB	−11.07	2P16	−8.65	2PHB	−11.63
11	2XBX	−10.33	2PHB	−10.40	2XBX	−11.90	2XBX	−10.82	2PHB	−8.62	2PHB	−11.54
12	2XBX	−10.26	2PHB	−10.38	3KL6	−11.89	2XBX	−10.82	2PHB	−8.43	2XBX	−11.28
13	2PHB	−9.86	2PHB	−10.36	2XBX	−11.80	2XBX	−10.76	2P16	−8.19	2PHB	−10.55
14	2PHB	−9.86	2XBX	−10.29	2P16	−11.80	3M36	−10.34	2XBX	−8.09	3M36	−10.45
15	2W26	−9.73	2XBX	−10.28	3KL6	−11.79	2XBX	−10.22	2PHB	−7.94	2XBX	−9.72
16	3M36	−9.70	3KL6	−10.21	2P16	−11.76	3M36	−10.18	2PHB	−7.59	2PHB	−8.72
17	3M36	−9.67	2XBX	−10.20	2PHB	−11.71	3M36	−10.11	2PHB	−7.49	2PHB	−8.64
18	2PHB	−9.66	2XBX	−10.18	2PHB	−11.66	2PHB	−10.11	2PHB	−7.41	2PHB	−8.23
19	2PHB	−9.65	2PHB	−10.17	2PHB	−11.42	2XBX	−10.09	2PHB	−7.32	2XBX	−8.08
20	2PHB	−9.62	2XBX	−10.14	2PHB	−11.40	2P16	−9.95	2PHB	−7.30	2XBX	−7.92
21	2P16	−9.59	3M36	−10.08	2PHB	−11.40	3KL6	−9.83	2XBX	−7.24	2PHB	−7.23
22	3KL6	−9.54	2PHB	−9.91	2PHB	−11.39	2XBX	−9.77	2PHB	−7.11	2PHB	−7.20
23	2XBX	−9.47	2P16	−9.88	2PHB	−11.27	2XBX	−9.59	2PHB	−7.05	2XBX	−7.19
24	2PHB	−9.44	2PHB	−9.64	2PHB	−11.18	2XBX	−9.48	3KL6	−6.98	2XBX	−5.35
25	2W26	−9.40	2PHB	−9.63	2PHB	−11.13	3KL6	−9.47	4ZAE	−6.86	4ZAE	−5.27
26	3KL6	−9.37	3M36	−9.58	3KL6	−11.12	3KL6	−9.39	2PHB	−6.74	4ZAE	−5.18
27	2W26	−9.37	3KL6	−9.46	2XBX	−11.01	2PHB	−9.28	2PHB	−6.70	4Z0K	−5.02
28	3KL6	−9.36	3KL6	−9.41	2XBX	−11.01	3KL6	−9.28	3KL6	−6.50	4ZAE	−4.98
29	3M36	−9.36	2XBX	−9.40	3KL6	−11.01	3KL6	−9.21	4ZAE	−6.47	4Z0K	−4.94
30	2PHB	−9.35	2PHB	−9.38	2PHB	−11.00	2P16	−9.11	2XBX	−6.44	4Z0K	−4.92

**Table 4:** The co-crystallized structures of protein–ligand complexes used in this work with their IC_50_ values, reference binding scores, reference binding energies, cognate docking scores and the corresponding RMSD values.

PDB ID	Resolution	Target	Ligand Name	hFIXa IC50 (nM)	hFXa IC50 (nM)	Score-In-Place	Cognate Docking	
						GScore (kcal/mol)	GScore (kcal/mol)	RMSD (Å)
5TNT	1.40 Å	hFIXa	CFM-184	4.90	31000.00	−9.78	−9.95	0.31
5TNO	1.54 Å		Selective ligand	98.00	>100000	−10.14	−9.38	0.90
5EGM	1.84 Å		Lead compound	0.60	234.00	−9.69	−9.85	1.22
4ZAE	1.86 Å		Lead compound	3.60	105.00	−9.64	not docked	-
4Z0K	1.41 Å		Lead compound	462.00	-	−10.30	−10.33	0.12
4YZU	1.41 Å		Inactive	9000.00	62500.00	−9.33	−9.71	0.99
2W26	2.08 Å	hFXa	Rivaroxaban	Selective	0.70	−9.65	−9.73	0.20
2P16	2.30 Å		Apixaban	Selective	0.08	−11.52	−11.48	0.46
3KL6	1.45 Å		Letaxaban	Selective	3.50	−11.58	−12.35	0.25
2PHB	2.30 Å		Eribaxaban	Selective	0.32	−11.18	−11.18	1.37
3M36	2.15 Å		DPC-423	Selective	0.15	−13.71	−13.78	0.00
2XBX	1.85 Å		Lead compound	-	15.00	−15.03	−14.83	1.41

## Data Availability

The data used to support the finding of this study are included in the Supplementary Materials.

## Supplementary Materials

The following are available online at https://www.mdpi.com/article/10.3390/molecules26175372/s1. Figure S1: The cascading mechanism of blood coagulation process; Figure S2: The overall structural deformations of the binding site of the two sets of proteins; Figure S3: Catalytic Triad; Figure S4: The conserved and non-conserved residues on the aligned sequences; Figure S5: The cognate docking poses; Figure S6: RMSD plot of all the FIXa and FXa proteins; Figure S7: The total energy of the complex; Figure S8: Radius of Gyration plot for FIXa and FXa; Figure S9: RMSF plot for FIXa and FXa, Table S1: Cross docking data with FIXa grids; Table S2: Cross docking data with FXa grids; Table S3: ADMET descriptors for twelve co-crystalized structures using QikProp.

## Sample Availability

Samples of the compounds are not available from the authors.
