# Supplementary material for: A Structure Based Study of Selective Inhibition of Factor IXa over Factor Xa
Source: Molecules. 2021 Sep 3;26(17):5372. doi: 10.3390/molecules26175372 (PMC8434132; doi:10.3390/molecules26175372)
Supplement: Supplementary file 1 [file molecules-26-05372-s001.zip › molecules-1287531-supplementary (1).pdf]

*molecules*

MDPI

*Supplementary Information*

# A Structure Based Study of Selective Inhibition of Factor IXa over Factor Xa

**Sibsankar Kundu [1] and Sangwook Wu [1,2,3,*]**

[1] Department of Physics, Pukyong National University, Busan 48513, Korea; current email: sskundu@pharmcadd.com
[2] R&D Center of PharmCADD, Busan 48060, Korea
[3] Department of Chemistry, University of North Carolina, Chapel Hill, NC 27599, USA
* Correspondence: sangwoow@pknu.ac.kr; Tel.: +82-051-629-5578

**Citation:** Kundu, S.; Wu, S. A Structure Based Study of Selective Inhibition of Factor IXa over Factor Xa. *Molecules* **2021**, *26*, 5372. https://doi.org/10.3390/molecules26175372

Academic Editor: George Grant; Alessandro Pedretti
Received: 21 June 2021
Accepted: 30 August 2021
Published: 3 September 2021

**Publisher's Note:** MDPI stays neutral with regard to jurisdictional claims in published maps and institutional affiliations.

**Copyright:** © 2021 by the authors. Licensee MDPI, Basel, Switzerland. This article is an open access article distributed under the terms and conditions of the Creative Commons Attribution (CC BY) license (http://creativecommons.org/licenses/by/4.0/).

## Coagulation Process

Blood coagulation is a complex physiological process that prevents bleeding due to the injury of the blood vessel by clot formation and maintains the normal flow of blood under the physiological condition. This process is known as hemostasis. This complex process of coagulation works through a series of cascading enzymatic reactions which involves a number of target proteins. Most of these target proteins are from a single subfamily (S1A) of serine protease and these are known as coagulation factors. The process is briefly described in Figure S1 to summarize the basic mechanism of coagulation. The coagulation process is subdivided into three smaller pathways, viz. intrinsic pathway, extrinsic pathway and common pathway. While the intrinsic pathway is triggered by blood vessel injury, the extrinsic pathway is triggered by tissue injury. The intrinsic pathway then propagates downstream through the conversion of FXII to FXIIa, FXI to FXIa and FIX to FIXa and the extrinsic pathway propagates downstream through the conversion of FVII to FVIIa with the help of tissue factor (TF). These two pathways are then merged to a common pathway which converts FX to FXa and propagates further downstream through the conversion of prothrombin to thrombin and finally to fibrillation or fibrin clot formation. FIXa and FXa are shown by red enclosing boxes in Figure S1 that describes how FIXa regulates the intrinsic pathway selectively without affecting the extrinsic pathway.

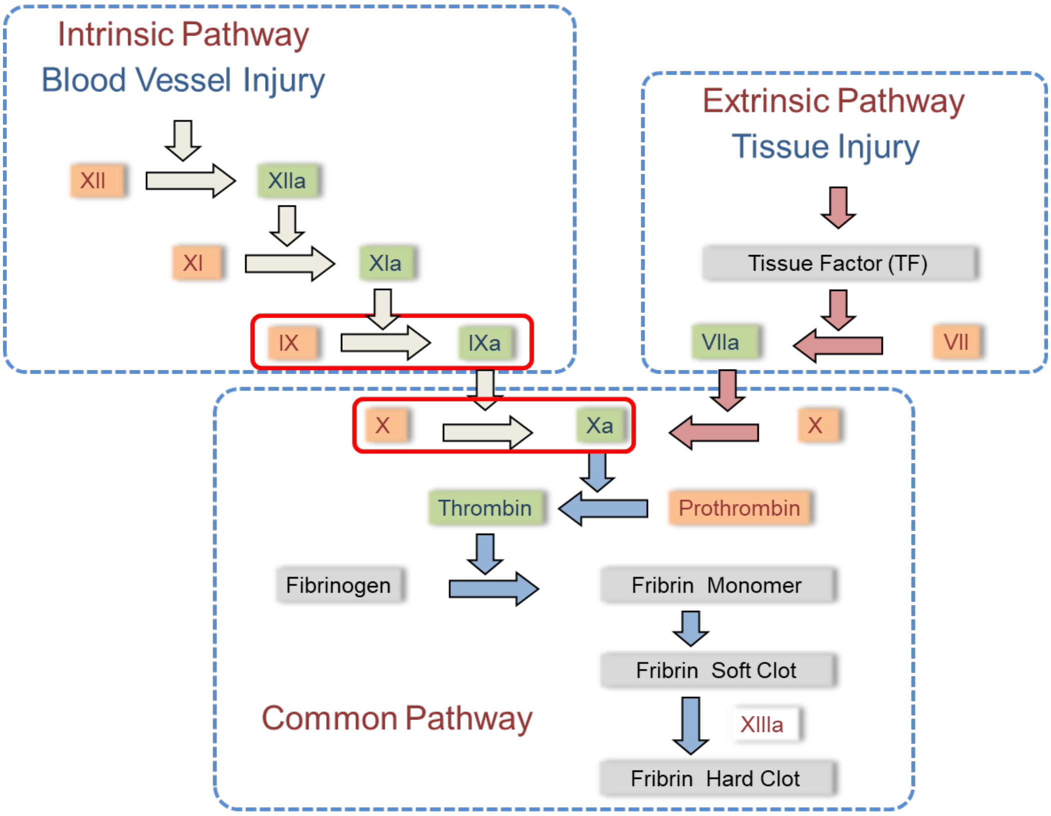


**Figure S1.** The cascading mechanism of blood coagulation process is shown with intrinsic pathway, extrinsic pathway and common pathway. The figure shows that FXa (Factor Xa) is in the common pathway and FIXa (Factor IXa) is under the intrinsic pathway (marked in red enclosing boxes). The activated factors are marked in green and the inactive factors are marked in orange.

The existing recent treatments are the direct inhibitors of FXa (Factor Xa) which falls under the common pathway. Therefore, the coagulation process is affected by both the intrinsic pathway and the extrinsic pathway. During the course of medication, in the event of severe injury, the bleeding risk would be a major issue due to the action of these anti-coagulants. Hence there is a need for therapeutic agents that can selectively regulate the intrinsic pathway without disturbing the normal regulation of the extrinsic pathway.

**Structural deformations of the binding site in the two sets of proteins**

Even though the binding site and binding mode of the ligands are very similar in the two sets of proteins, there exist a small structural deformations in these two sets of proteins. The structural deformation of the ligand binding site occurs in these two sets of proteins mainly due to two reasons—(a) the changes induced by the different ligands within the same protein either in FIXa or FXa, and (b) the changes in the amino acid sequence in these two sets of proteins, i.e., between the protein FIXa and protein FXa. With all the causes the overall structural deformations around the binding sites are very minor except few residues or regions. This was shown in the following Figure S2. Ligands binding to the active site often induces deformations of the active sites. These deformations occur through the displacements of the side chains and sometimes the backbone of the proteins. In order to visualize these small structural changes, all the proteins are superposed and depicted in Figure S2. This comparison is proper within the same proteins (with identical primary sequence) bound to different ligands. In case of different proteins (where primary sequence is different in the two proteins) we compare the conserved residues within the two sets of proteins.

- Structural alignment of all the FIXa proteins to understand the ligand induced deformations, Figure S2 (b).

- Structural alignment of all the FXa proteins to understand the ligand induced deformations, Figure S2 (d).
- Both the set of proteins together in different color and superposed to understand overall deformations, Figure S2 (f).

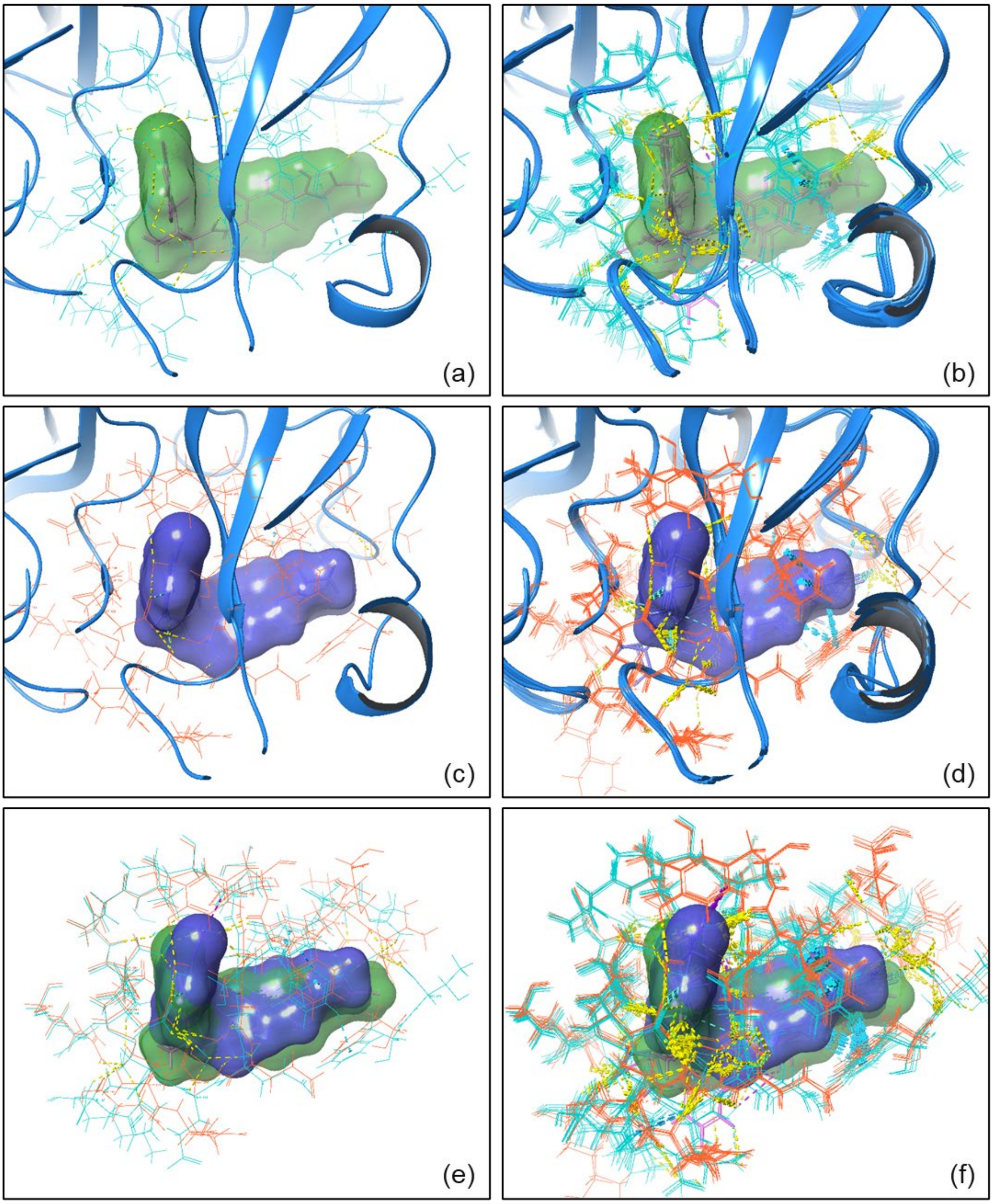


**Figure S2.** The overall structural deformations of the binding site of the two sets of proteins are shown from behind, in order to make most of the residues visible. (**a**) for the protein FIXa with one protein (PDB ID: 5TNT) and (**b**) for all the proteins of FIXa to represent the deformations of the binding site residues due to the binding of various ligands. (**c**) for the protein FXa with one protein (PDB ID: 2W26) and (**d**) for all the proteins of FXa to represent the deformations of the binding site residues due to the binding of various ligands. (**e**) the superposition of (**a**) and (**c**) and (**f**) is the superposition of (**b**) and (**d**).

**Binding Pocket and Binding Mode Description**

The substrate binding pocket of FIXa and FXa have significant similarities. It consists of a set of conserved residues and a set of non-conserved residues. The overall structural alignment of the binding site of FIXa and FXa are shown in Figure 1 (in the original article), where it is visible that there are minor changes around the S1 pocket in these two sets of proteins, but there are significant changes around the S3 and S4 pockets in some cases. The residues where the changes are significant are analyzed with further details and discussed in the later part of this section. It is observed from the aligned sequence that there are several insertions and deletions in the two protein sequences, FIXa and FXa, which is located in the S4 pocket of the active site of FIXa and FXa within 3 Å distance. These changes in the number of residues make the S4 pocket of FIXa slightly different than the S4 pocket in FXa. This fact has been investigated in more detail using various docking experiments described in the following subsections. The binding mode which is the orientation of the ligand along the binding site is identical in both FIXa and FXa proteins. All the co-crystal ligands are oriented identically in an "L-shape" in both the proteins, i.e., the shorter side of the "L" and the longer side of the "L" are oriented in the similar way which is shown in Figure 1a and 1b. The multiple sequence alignment (MSA) was done for these two sets of proteins and analyzed to establish the role of conserved and non-conserved residues that contributes to the ligand selectivity in these two sets of proteins (Figure 2). The dots in the multiple sequence alignment (MSA) represent the residue identities. Interestingly, though there are significant differences in sequences between these two sets (FIXa and FXa) of proteins, the functional architecture, binding site residues, binding mode of the ligands in these two sets of proteins are very similar (Figure 1a, 1b and 1c). Being in the same subfamily (S1A) of proteins, they have conserved functional architecture. The aligned sequences and the corresponding structural changes have been analyzed for various regions in the binding site that are critical for their selectivity. These regions are catalytic triad and the various subsites of the substrate binding site and they are also sometimes called pockets throughout this article, viz. S1, S2, S3 and S4. The sequence of the proteins, that are bound to various potent and selective ligands of the same target, are compared and identify the residues that are in close proximity to the ligands in the binding site. The corresponding 2D ligand interaction diagram (LID) for all the structures shown in Figure 3(for FIXa) and Figure 4 (for FXa) with 4 Å distance from the ligands.

Catalytic Triad—The catalytic site is located at the heavy chain and the catalytic triad consists of three residues – Ser195, His57 and Asp102. Among the 3 residues, the only residue that is at the binding site and within the 4 Å of the ligand (in all cases) is Ser195 (Figure S4). This activates Ser195 by charge relay through the H-bond network. His57 is within 5 Å distance of ligands for half of the proteins (only one within 3 Å distance, in 5EGM). The residue Asp102 is beyond 5 Å distance of the ligand in all the cases. Therefore, this triad sets a communication chain from the active sites to the other part of the protein. It is observed that Ser195, His57 and Asp102 are conserved over the two sets of proteins. These three residues in the catalytic triad are structurally conserved as shown in Figure S3(a). Figure S3(b) shows that there is another residue, Ser190 (in FIXa) and Aal190 (in FXa), which is very close to this catalytic tried and structurally conserved.

S1 Packet—S1 pocket consists of Trp215, Gly216 and Glu217, Glu219 (FIXa) and Gly219 (FXa) on one side and Asp189, Ser190 (in FIXa) and Ala190 (in FXa), Cys191 and Gln192 on the other side. Most of these residues are conserved in these two sets of proteins except the residue Ser190 (in FIXa)/ Ala190 (in FXa) and Glu219 (FIXa)/ Gly219 (FXa). These two residues play an important role since they are different in nature. While Glu and Ser are charged and polar residues respectively, Gly and Ala are hydrophobic in nature. The bottom of the S1 pocket (which is buried deeper inside) is lined up by Asp189 and Tyr228 and both the residues are conserved in these two sets of proteins, while Asp189 is mostly within 3 Å and 4 Å distance (with one exception in 5EGM) but Tyr228 is within 4 Å, 5 Å and beyond.

The region of the S1 pocket that starts from Asp189 and ends to Ser195 are shown on the aligned sequence in Figure S4 and the corresponding structural alignments are shown in Figure S4(b) (front view) and Figure S4(a) (side view). In this region, only one difference is Ser190 (FIXa) and Ala190 (FXa) (Figure S4(c)). This residue is always within 3 Å distance of the ligand and sometimes forms H-bond between backbone > CO (carbonyl) for both sets of proteins. Ser190 being within 3 Å distance forms another H-bond with FIXa-ligands, which is not possible in the case of FXa-ligands.

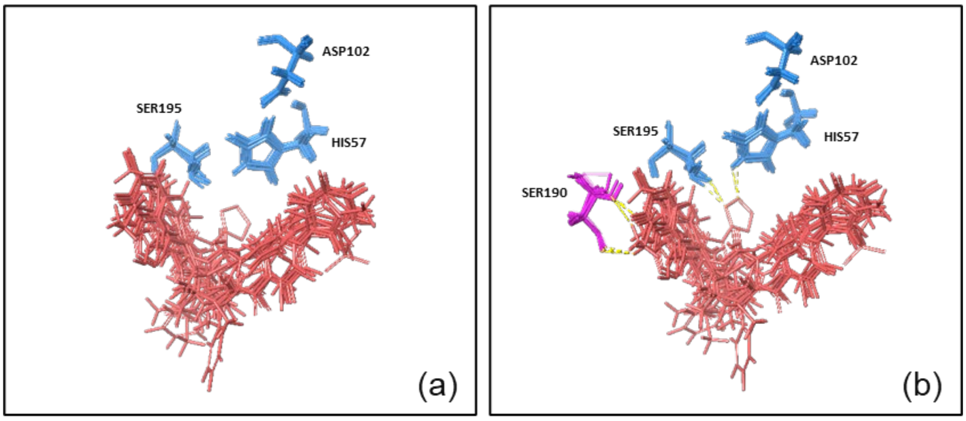


**Figure S3.** (**a**) The three residues, His57, Asp102, Ser195, that forms the catalytic triad, are structurally conserved. Ser195 interacts through the H-bond. (**b**) There is another Ser190 (in FIXa) and Ala190 (in FXa) which is in the S1 pocket and is close to this catalytic triad.

Gly219 bridges the two pockets, S1 and S3, of the active site (Figure S4(d)), whereas in FIXa the Glu219 is somewhat away from the active site and cannot bridge the two sides of the L-shaped molecule (Figure S4(e) and Figure S4(f)). In the seven residues long fragment, Ile213-Cys220, there is one significant difference in the nature of residue, i.e. Glu219 (in FIXa) and Gly219 (in FXa). Therefore, FIXa active site can accommodate a bigger and a polar group since Glu219 is present (shown in Figure S4(f)), while the FXa preferred the small hydrophobic group in that place.

S3 and S4 Pocket—S3 and S4 pocket is surrounded mostly by the aromatic and hydrophobic residues, viz. Tyr99, Phe174 and Trp215. All three residues are conserved within the 3 Å distance of the ligand with one exception as Tyr99 is within 4 Å in 5EGM (PDB ID). This region is one of the most important regions that might play a role in the selectivity of binding. Tyr99 is conserved over all the proteins FXa and FIXa (shown in Figure S4(g)). But there are few insertions in FIXa in this region shown in Figure S4(h) (front view) and Figure S4(i) (side view). All these residues together make the binding site a bit compact and tight for FXa as compared to FIXa. In order to perform the structure and sequence analysis, it is needless to mention that within the 5 Å proximity of the ligand, there are some residues that are conserved and some of the residues which are different. The most critical residues for selectivity are those which belong to the active site or binding site and are different within the two proteins.

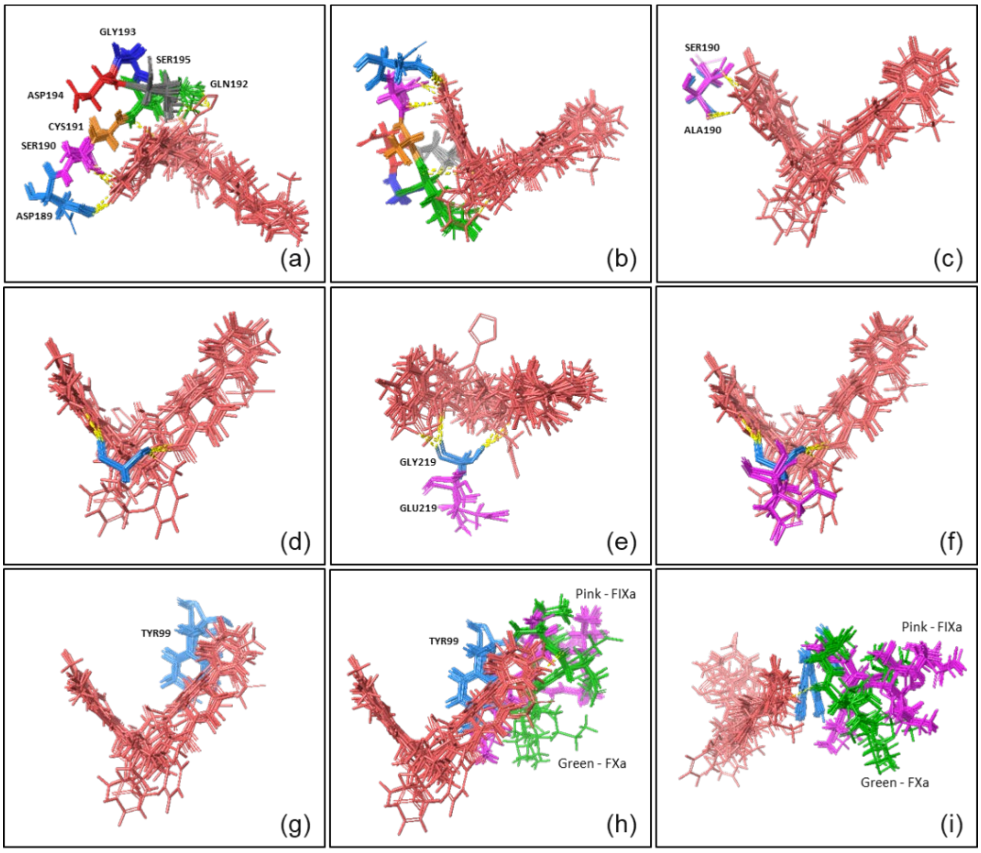


**Figure S4.** The conserved and non-conserved residues on the aligned sequences are shown in the 3D structure. They show structural differences in the binding pocket. The residues within the 3 Å distance of the ligands mostly play important role in the binding process. The ligands are shown in brown. (**a**) The structural alignment of residues from Asp189 (azure blue) to Ser195 (gray) are shown for all the proteins as a side view and the front view is shown in (**b**). (**c**) There is only one difference in this segment of residues between FIXa and FXa, which is Ser190 (in FIXa) (pink) and Ala190 (FXa) (azure blue). While Ser190 is forming an H-bond in FIXa, the same is absent in FXa with Ala190. (**d**) Gly219 (azure blue) forms H-bonds to bridge the S1 and S3 pockets in FXa. (**e**) Glu219 (pink) in FIXa is pushed away from the binding site without making any H-bond (side view). (**f**) Gly219 (FXa) and Glu219 (FIXa) are shown in a single frame (front view). (**g**) Tyr99 (azure blue) is conserved in all the proteins. (**h**) Ala95 to Lys98 in FIXa (pink) and Thr95 to Thr98 in FXa (pink) while the Tyr99 is conserved and shown in azure blue (front view). (**i**) The same as (**h**) as a side view.

## Cognate docking pose comparison and RMSD

The RMSD is calculated between docked pose (after re-docking) and the crystal ligand and it is observed that the RMSD values are very low with few exceptions. The structural superposition of the docked poses and the crystal ligands are shown in Figure S5 and the RMSD values are given in the caption of the figure (and also in Table 4 of the main article).

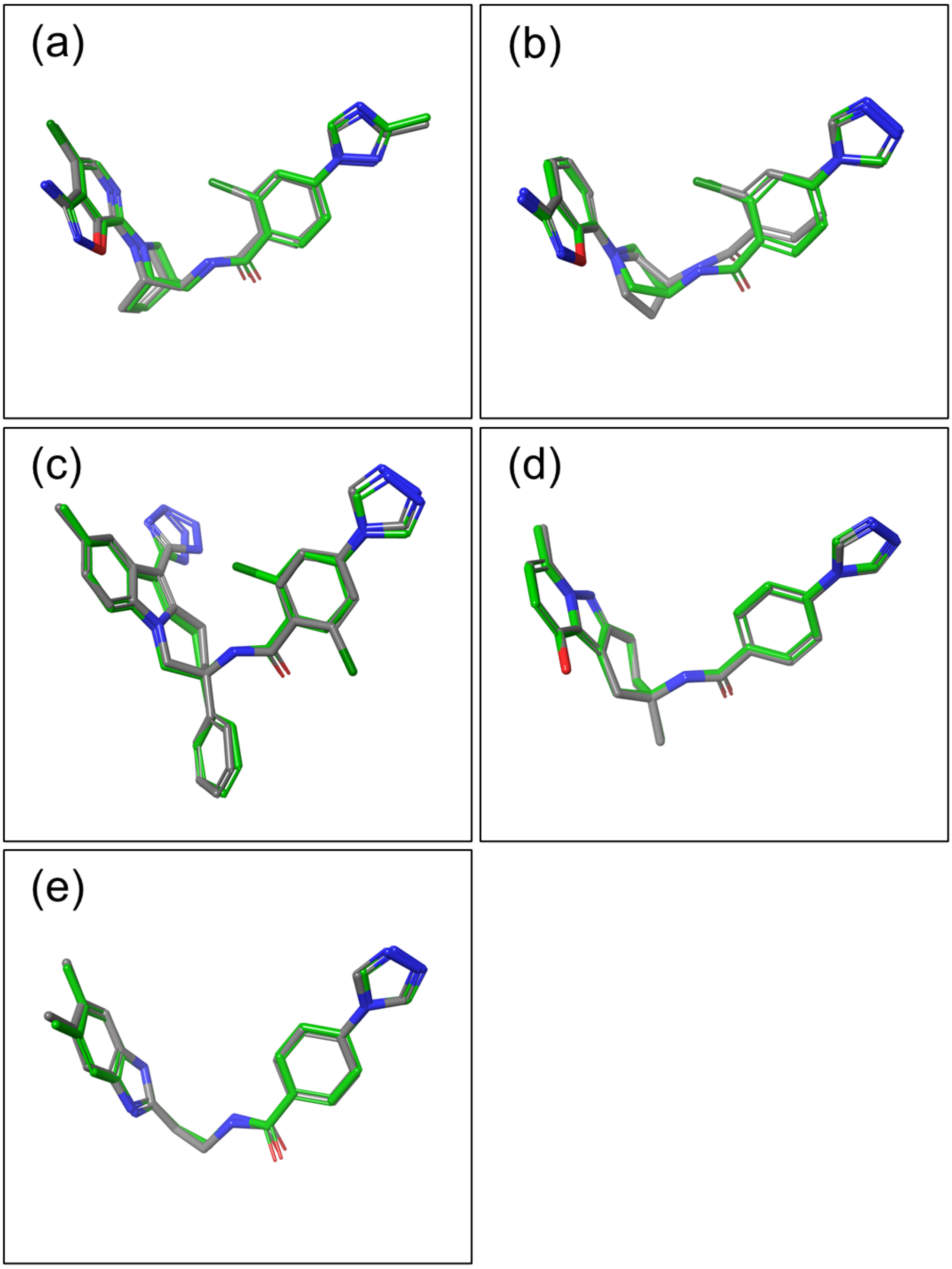


**Figure S5.** Continued on next page.

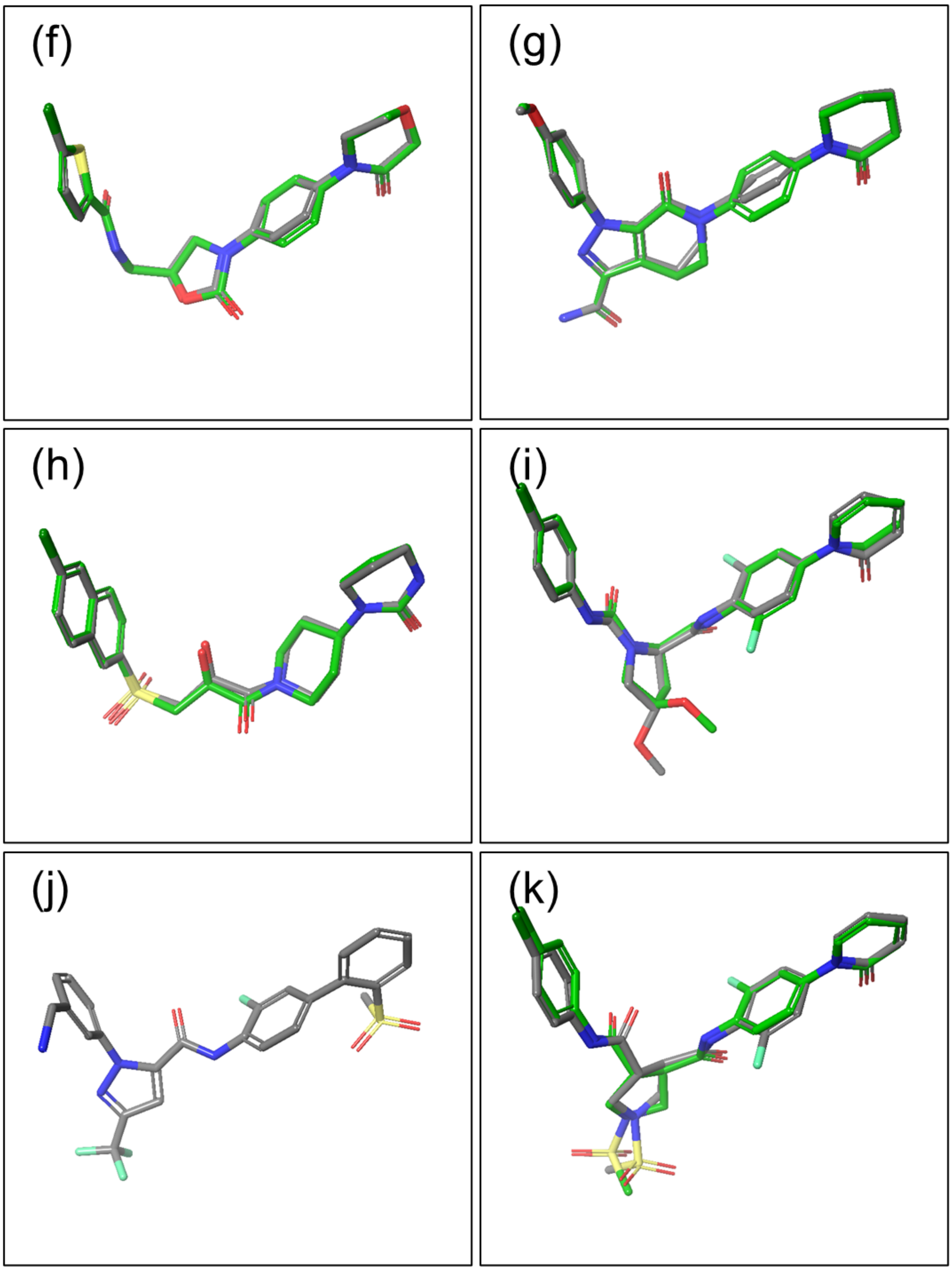


**Figure S5.** The cognate docking poses are compared with the corresponding co-crystal ligands and the RMSD is calculated for all the heavy atoms of FIXa and FXa proteins, listed in Table 4. (**a**) 5TNT (RMSD = 0.31 Å), (**b**) 5TNO (RMSD = 0.90 Å), (**c**) 5EGM (RMSD = 1.22 Å), (**d**) 4Z0K (RMSD = 0.12 Å), (**e**) 4YZU (RMSD = 0.99 Å), (**f**) 2W26 (RMSD = 0.20 Å), (**g**) 2P16 (RMSD = 0.46 Å), (**h**) 3KL6 (RMSD = 0.25 Å), (**i**) 2PHB (RMSD = 1.37 Å), (**j**) 3M36 (RMSD = 0.00 Å) and (**k**) 2XBX (RMSD = 1.41 Å).

**Molecular Dynamic Simulation**

Molecular Dynamic (MD) simulations have been performed to evaluate the stability of the protein-ligand complexes using Gromacs 2019 software[1]. Amber99sb-ildn force field [2] has been used for the protein and the ligand parameters are generated using the program Antechamber [3] with BCC-AM1 charge set. The protein-ligand complex was

placed in a cubic box and solvated with explicit water (TIP3 water model) layer that extends 0.1nm from the protein in all direction. The solvated protein-ligand system was neutralized with 0.1M salt concentration of NaCl. The system was minimized and equilibrated using NVT and NPT protocol for 1ns at 300K temperature. Throughout the equilibrium process system was maintained with restrained state. Final production run was performed for 100ns in triplicate for all twelve proteins (6 for FIXa and 6 for FXa).

The stability of the protein-ligand system was evaluated in terms of RMSD, total energy, radius of gyration and RMSF and they are plotted with time. The figures below shows that the protein-ligand complexes are stable throughout the dynamics. In all the following figures there are two frames, (a) and (b). Frame (a) plots all the FIXa protein data and frame (b) plots all the FXa protein data with the same order and the same color schemes in all figures.

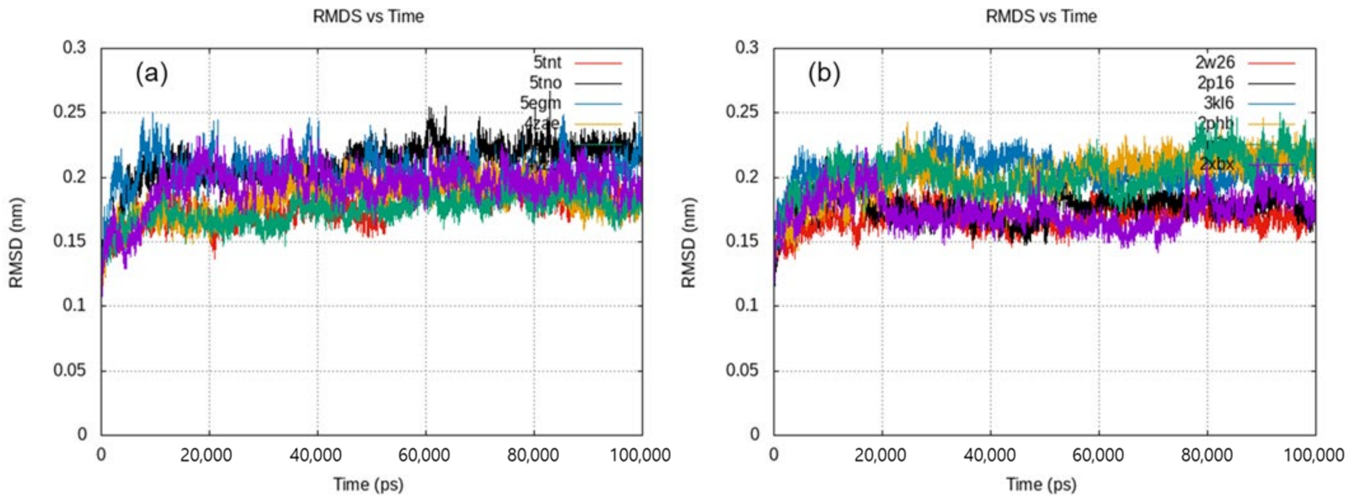


**Figure S6.** RMSD plot of (**a**) all the FIXa and (**b**) FXa proteins show that the structure of the protein is not changing much throughout the dynamics.

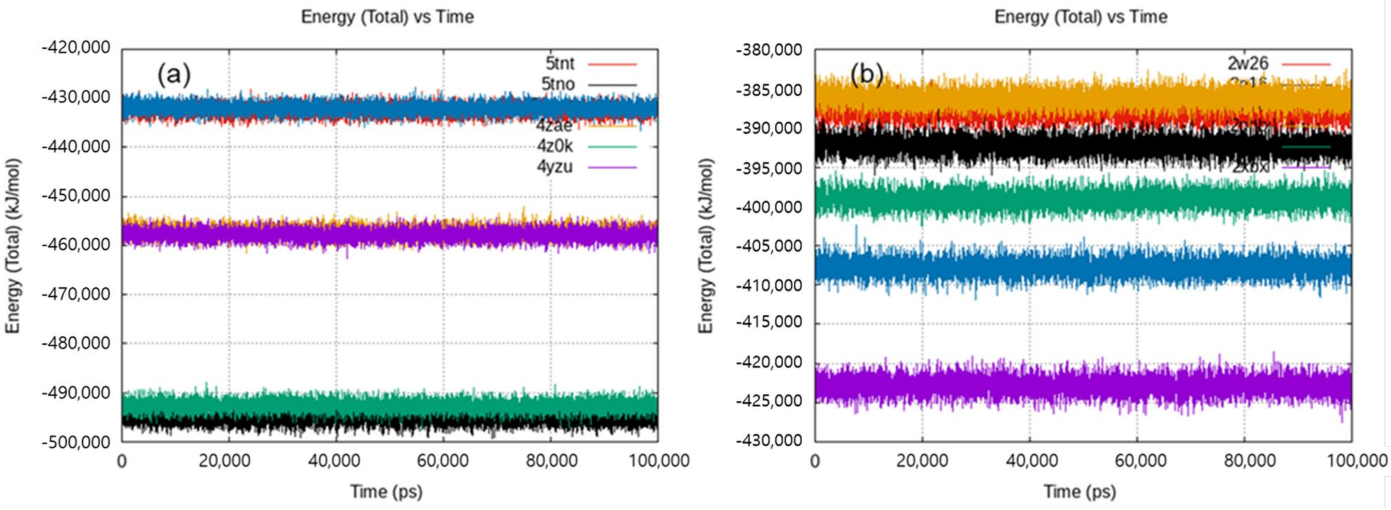


**Figure S7.** The total energy of the complex is plotted against time in (**a**) for FIXa proteins and (**b**) for FXa proteins. Even though the total energy of different protein-ligand complex varies in absolute values, but in a particular protein-ligand system the energy varies in a very small range around the mean value. This indicates the stability of the protein-ligand system throughout the dynamics.

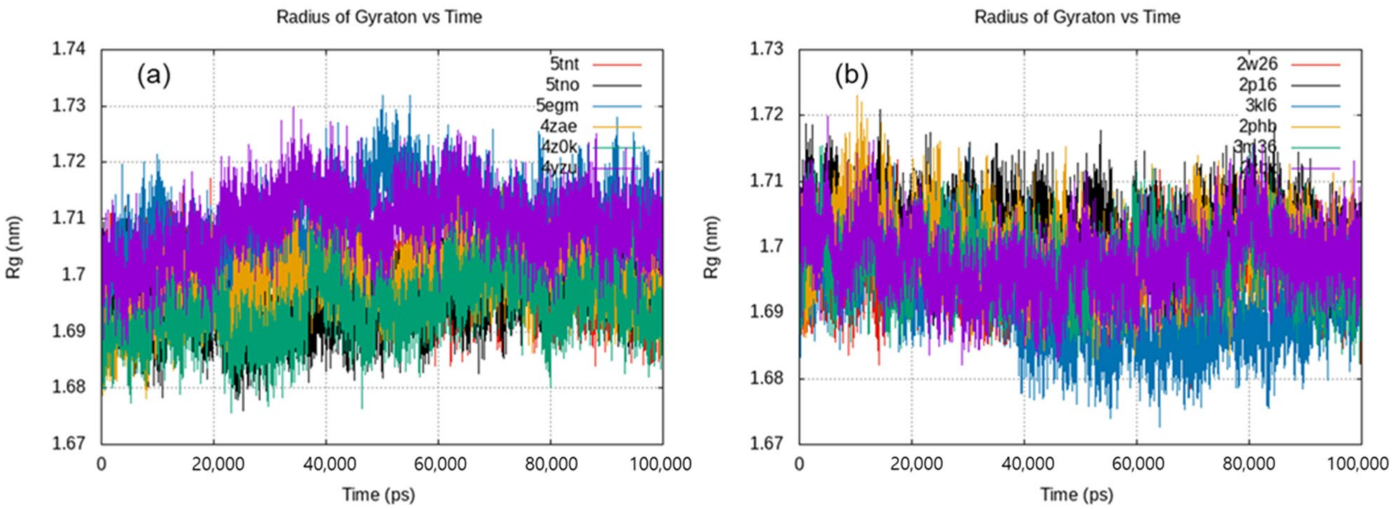


**Figure S8.** Radius of Gyration plot for (**a**) FIXa and (**b**) FXa.

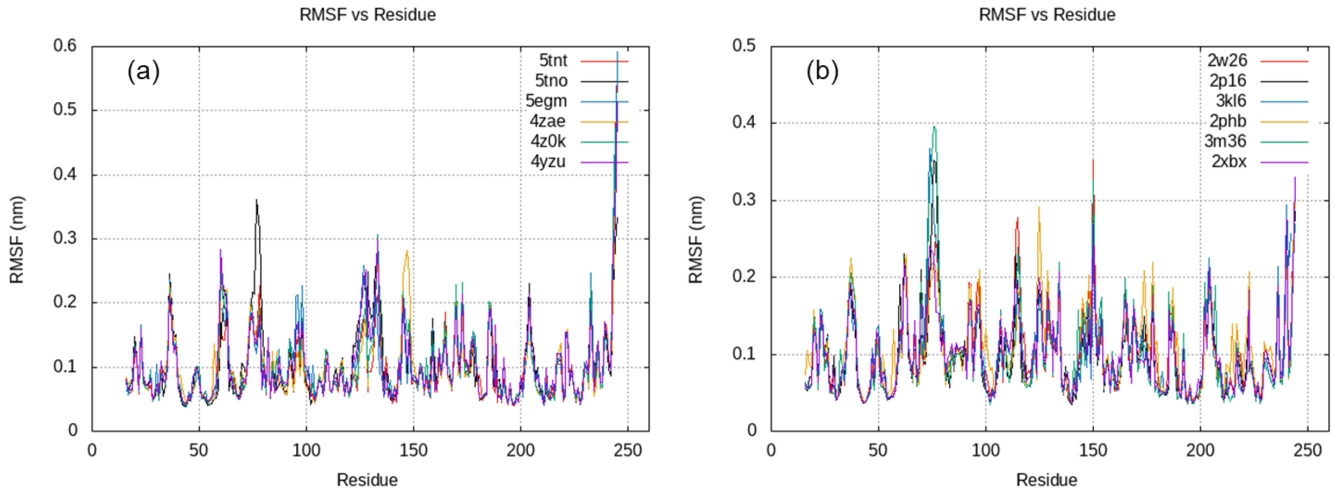


**Figure S9.** RMSF plot for (**a**) FIXa and (**b**) FXa.

**Table S1.** Cross docking data with FIXa grids.

| | FIXa | | | | | | | | | | | |
|---|---|---|---|---|---|---|---|---|---|---|---|---|
| | Grid 5TNT | | Grid 5TNO | | Grid 5EGM | | Grid 4ZAE | | Grid 4Z0K | | Grid 4YZU | |
| | | Gscore (kcal/mol) | | Gscore (kcal/mol) | | GScore (kcal/mol) | | GScore (kcal/mol) | | GScore (kcal/mol) | | GScore (kcal/mol) |
| 1 | 5TNT | −9.95 | 5TNT | −9.65 | 5EGM | −9.85 | 2XBX | −9.86 | 4Z0K | −10.33 | 4YZU | −9.71 |
| 2 | 5TNT | −9.77 | 5TNO | −9.38 | 5EGM | −9.79 | 3KL6 | −9.56 | 4Z0K | −9.50 | 4YZU | −9.63 |
| 3 | 5TNT | −9.60 | 5TNT | −9.18 | 5EGM | −9.17 | 2XBX | −9.41 | 5TNT | −8.81 | 3KL6 | −9.49 |
| 4 | 2XBX | −8.29 | 5TNO | −8.89 | 4Z0K | −8.45 | 3M36 | −9.35 | 5TNO | −8.81 | 4ZAE | −9.29 |
| 5 | 3KL6 | −8.17 | 5TNO | −8.58 | 2XBX | −8.15 | 2XBX | −9.21 | 2XBX | −8.54 | 3KL6 | −9.23 |
| 6 | 2XBX | −8.06 | 4Z0K | −8.58 | 2XBX | −8.12 | 2XBX | −9.15 | 5TNO | −8.52 | 2XBX | −9.09 |
| 7 | 2XBX | −7.89 | 2XBX | −8.54 | 2XBX | −8.10 | 2XBX | −9.06 | 5TNO | −8.50 | 3KL6 | −9.03 |
| 8 | 2PHB | −7.81 | 4Z0K | −8.28 | 4Z0K | −8.04 | 2PHB | −8.97 | 4YZU | −8.36 | 3KL6 | −8.98 |
| 9 | 2PHB | −7.70 | 2W26 | −8.07 | 2XBX | −8.01 | 2XBX | −8.90 | 5TNO | −8.30 | 4YZU | −8.96 |

| | | | | | | | | | | | | |
|---|---|---|---|---|---|---|---|---|---|---|---|---|
| 10 | 2XBX | −7.68 | 3KL6 | −7.95 | 2XBX | −7.88 | 3M36 | −8.89 | 5TNO | −8.20 | 4ZAE | −8.96 |
| 11 | 2XBX | −7.63 | 5TNT | −7.84 | 2XBX | −7.86 | 2XBX | −8.80 | 2XBX | −7.73 | 4YZU | −8.85 |
| 12 | 2XBX | −7.60 | 2PHB | −7.83 | 2W26 | −7.85 | 3KL6 | −8.72 | 4YZU | −7.73 | 3KL6 | −8.81 |
| 13 | 2PHB | −7.57 | 2PHB | −7.82 | 2PHB | −7.60 | 2PHB | −8.71 | 2XBX | −7.68 | 2XBX | −8.73 |
| 14 | 2PHB | −7.53 | 2XBX | −7.78 | 2PHB | −7.49 | 2XBX | −8.71 | 2XBX | −7.66 | 2PHB | −8.70 |
| 15 | 2XBX | −7.47 | 2PHB | −7.68 | 2PHB | −7.47 | 2XBX | −8.66 | 2XBX | −7.61 | 2XBX | −8.62 |
| 16 | 2XBX | −7.47 | 3KL6 | −7.68 | 2W26 | −7.45 | 3KL6 | −8.65 | 2XBX | −7.56 | 2XBX | −8.48 |
| 17 | 2XBX | −7.37 | 2PHB | −7.68 | 2PHB | −7.35 | 3M36 | −8.62 | 5TNO | −7.52 | 2XBX | −8.48 |
| 18 | 2PHB | −7.36 | 2XBX | −7.65 | 2W26 | −7.11 | 3M36 | −8.62 | 2W26 | −7.37 | 3KL6 | −8.47 |
| 19 | 2PHB | −7.35 | 2W26 | −7.62 | 2PHB | −7.05 | 5TNT | −8.61 | 2PHB | −7.35 | 2W26 | −8.45 |
| 20 | 3KL6 | −7.34 | 2XBX | −7.60 | 2PHB | −7.03 | 3KL6 | −8.57 | 2XBX | −7.32 | 3KL6 | −8.39 |
| 21 | 2XBX | −7.33 | 2PHB | −7.58 | 2PHB | −6.99 | 2XBX | −8.54 | 2XBX | −7.24 | 2XBX | −8.39 |
| 22 | 3KL6 | −7.30 | 2PHB | −7.54 | 2W26 | −6.98 | 2PHB | −8.49 | 2XBX | −7.19 | 3KL6 | −8.39 |
| 23 | 3KL6 | −7.21 | 2XBX | −7.46 | 2XBX | −6.98 | 2PHB | −8.46 | 3KL6 | −7.18 | 2W26 | −8.37 |
| 24 | 2PHB | −7.20 | 2PHB | −7.41 | 2W26 | −6.97 | 2P16 | −8.43 | 2W26 | −7.13 | 2W26 | −8.30 |
| 25 | 3KL6 | −7.13 | 3KL6 | −7.37 | 2PHB | −6.93 | 2PHB | −8.43 | 3KL6 | −7.13 | 2XBX | −8.29 |
| 26 | 2PHB | −7.07 | 3KL6 | −7.33 | 5TNT | −6.87 | 5TNT | −8.40 | 3KL6 | −7.06 | 3KL6 | −8.29 |
| 27 | 2XBX | −7.04 | 2XBX | −7.33 | 2PHB | −6.72 | 2PHB | −8.40 | 2PHB | −7.00 | 4ZAE | −8.19 |
| 28 | 2XBX | −7.04 | 2PHB | −7.31 | 2PHB | −6.63 | 2XBX | −8.40 | 2W26 | −6.99 | 2XBX | −8.16 |
| 29 | 2PHB | −6.85 | 2PHB | −7.31 | 2PHB | −6.59 | 3KL6 | −8.39 | 3KL6 | −6.97 | 2XBX | −8.00 |
| 30 | 3KL6 | −6.83 | 2XBX | −7.27 | 2W26 | −6.45 | 3KL6 | −8.31 | 2PHB | −6.95 | 2XBX | −7.93 |
| 31 | 2PHB | −6.82 | 3KL6 | −7.24 | 2XBX | −6.44 | 3KL6 | −8.27 | 3KL6 | −6.92 | 2XBX | −7.92 |
| 32 | 2PHB | −6.79 | 2W26 | −7.20 | 4Z0K | −6.41 | 3KL6 | −8.23 | 2W26 | −6.91 | 3KL6 | −7.90 |
| 33 | 2P16 | −6.62 | 2PHB | −7.12 | 2XBX | −6.29 | 3KL6 | −8.22 | 3KL6 | −6.87 | 2W26 | −7.87 |
| 34 | 3KL6 | −6.61 | 2W26 | −7.09 | 4YZU | −6.24 | 3KL6 | −8.19 | 2PHB | −6.84 | 2PHB | −7.83 |
| 35 | 3KL6 | −6.60 | 3KL6 | −7.07 | 2PHB | −6.24 | 2P16 | −8.16 | 3KL6 | −6.84 | 2PHB | −7.77 |
| 36 | 3KL6 | −6.54 | 2P16 | −7.00 | 5EGM | −6.10 | 2PHB | −8.08 | 3KL6 | −6.83 | 2W26 | −7.71 |
| 37 | 2PHB | −6.52 | 2W26 | −7.00 | 2XBX | −6.09 | 2PHB | −7.91 | 2XBX | −6.82 | 2W26 | −7.63 |
| 38 | 3KL6 | −6.31 | 2XBX | −7.00 | 2XBX | −5.98 | 2PHB | −7.89 | 2PHB | −6.74 | 3M36 | −7.62 |
| 39 | 3KL6 | −5.93 | 2PHB | −6.98 | 5EGM | −5.94 | 2PHB | −7.89 | 3KL6 | −6.68 | 4ZAE | −7.56 |
| 40 | 2P16 | −5.91 | 2PHB | −6.98 | 4YZU | −5.92 | 2XBX | −7.84 | 2XBX | −6.68 | 2XBX | −7.54 |
| 41 | 3M36 | −5.40 | 2XBX | −6.94 | 3M36 | −5.92 | 2P16 | −7.73 | 2PHB | −6.65 | 2PHB | −7.52 |
| 42 | 5EGM | −5.29 | 2W26 | −6.92 | 4YZU | −5.74 | 2PHB | −7.72 | 2PHB | −6.56 | 2PHB | −7.51 |
| 43 | 3M36 | −5.20 | 3KL6 | −6.85 | 4YZU | −5.57 | 2PHB | −7.64 | 2PHB | −6.49 | 4ZAE | −7.43 |
| 44 | 3KL6 | −5.14 | 3KL6 | −6.84 | 5EGM | −5.46 | 5TNT | −7.51 | 2XBX | −6.41 | 2PHB | −7.41 |
| 45 | 5EGM | −5.14 | 3KL6 | −6.79 | 3M36 | −5.38 | 3M36 | −7.20 | 2PHB | −6.40 | 2PHB | −7.37 |
| 46 | 5EGM | −5.05 | 3M36 | −6.73 | 3M36 | −5.36 | 3M36 | −7.01 | 4ZAE | −6.34 | 3KL6 | −7.33 |
| 47 | 5TNO | −4.96 | 2P16 | −6.60 | 4YZU | −5.35 | 3KL6 | −6.96 | 2PHB | −6.32 | 2PHB | −7.25 |
| 48 | 3M36 | −4.94 | 4Z0K | −6.59 | 4YZU | −5.19 | 3KL6 | −6.84 | 2PHB | −6.28 | 4ZAE | −7.22 |
| 49 | 5TNO | −4.92 | 3KL6 | −6.59 | 3M36 | −5.10 | 5TNO | −6.47 | 2W26 | −6.28 | 2PHB | −7.04 |
| 50 | 2P16 | −4.82 | 3KL6 | −6.59 | 5TNO | −4.98 | 5TNO | −6.29 | 2W26 | −6.24 | 3M36 | −7.00 |

**Table S2.** Cross docking data with FXa grids.

| | FXa | | | | | | | | | | | |
|---|---|---|---|---|---|---|---|---|---|---|---|---|
| | Grid 2W26 | | Grid 2P16 | | Grid 3KL6 | | Grid 2PHB | | Grid 3M36 | | Grid 2BXB | |
| | | GScore (kcal/mol) | | GScore (kcal/mol) | | GScore (kca/mol) | | GScore (kcal/mol) | | GScore (kcal/mol) | | GScore (kcal/mol) |
| 1 | 3M36 | −11.46 | 2P16 | −11.48 | 2XBX | −12.95 | 2XBX | −11.90 | 3M36 | −13.78 | 2XBX | −14.83 |
| 2 | 2XBX | −11.00 | 2XBX | −11.07 | 2XBX | −12.61 | 2XBX | −11.87 | 3M36 | −13.73 | 2XBX | −14.81 |
| 3 | 2PHB | −10.91 | 2XBX | −11.03 | 2XBX | −12.56 | 3M36 | −11.85 | 3M36 | −12.41 | 2XBX | −14.58 |
| 4 | 2XBX | −10.89 | 2XBX | −11.02 | 2XBX | −12.55 | 3M36 | −11.69 | 3M36 | −12.34 | 2PHB | −14.26 |
| 5 | 3M36 | −10.85 | 2XBX | −11.01 | 3KL6 | −12.35 | 2XBX | −11.64 | 3M36 | −11.65 | 2PHB | −14.09 |
| 6 | 2XBX | −10.80 | 3M36 | −10.73 | 2PHB | −12.28 | 2XBX | −11.52 | 3M36 | −11.35 | 2PHB | −13.00 |
| 7 | 2PHB | −10.73 | 3M36 | −10.65 | 2XBX | −12.27 | 3M36 | −11.49 | 2P16 | −9.06 | 2PHB | −12.86 |
| 8 | 3M36 | −10.72 | 2P16 | −10.63 | 3KL6 | −12.14 | 2PHB | −11.18 | 2XBX | −9.00 | 3M36 | −12.68 |
| 9 | 2XBX | −10.47 | 2PHB | −10.47 | 2P16 | −12.11 | 2PHB | −11.08 | 2XBX | −8.75 | 2XBX | −11.65 |
| 10 | 2P16 | −10.34 | 2PHB | −10.42 | 2PHB | −11.96 | 2PHB | −11.07 | 2P16 | −8.65 | 2PHB | −11.63 |
| 11 | 2XBX | −10.33 | 2PHB | −10.40 | 2XBX | −11.90 | 2XBX | −10.82 | 2PHB | −8.62 | 2PHB | −11.54 |
| 12 | 2XBX | −10.26 | 2PHB | −10.38 | 3KL6 | −11.89 | 2XBX | −10.82 | 2PHB | −8.43 | 2XBX | −11.28 |
| 13 | 2PHB | −9.86 | 2PHB | −10.36 | 2XBX | −11.80 | 2XBX | −10.76 | 2P16 | −8.19 | 2PHB | −10.55 |
| 14 | 2PHB | −9.86 | 2XBX | −10.29 | 2P16 | −11.80 | 3M36 | −10.34 | 2XBX | −8.09 | 3M36 | −10.45 |
| 15 | 2W26 | −9.73 | 2XBX | −10.28 | 3KL6 | −11.79 | 2XBX | −10.22 | 2PHB | −7.94 | 2XBX | −9.72 |
| 16 | 3M36 | −9.70 | 3KL6 | −10.21 | 2P16 | −11.76 | 3M36 | −10.18 | 2PHB | −7.59 | 2PHB | −8.72 |
| 17 | 3M36 | −9.67 | 2XBX | −10.20 | 2PHB | −11.71 | 3M36 | −10.11 | 2PHB | −7.49 | 2PHB | −8.64 |
| 18 | 2PHB | −9.66 | 2XBX | −10.18 | 2PHB | −11.66 | 2PHB | −10.11 | 2PHB | −7.41 | 2PHB | −8.23 |
| 19 | 2PHB | −9.65 | 2PHB | −10.17 | 2PHB | −11.42 | 2XBX | −10.09 | 2PHB | −7.32 | 2XBX | −8.08 |
| 20 | 2PHB | −9.62 | 2XBX | −10.14 | 2PHB | −11.40 | 2P16 | −9.95 | 2PHB | −7.30 | 2XBX | −7.92 |
| 21 | 2P16 | −9.59 | 3M36 | −10.08 | 2PHB | −11.40 | 3KL6 | −9.83 | 2XBX | −7.24 | 2PHB | −7.23 |
| 22 | 3KL6 | −9.54 | 2PHB | −9.91 | 2PHB | −11.39 | 2XBX | −9.77 | 2PHB | −7.11 | 2PHB | −7.20 |
| 23 | 2XBX | −9.47 | 2P16 | −9.88 | 2PHB | −11.27 | 2XBX | −9.59 | 2PHB | −7.05 | 2XBX | −7.19 |
| 24 | 2PHB | −9.44 | 2PHB | −9.64 | 2PHB | −11.18 | 2XBX | −9.48 | 3KL6 | −6.98 | 2XBX | −5.35 |
| 25 | 2W26 | −9.40 | 2PHB | −9.63 | 2PHB | −11.13 | 3KL6 | −9.47 | 4ZAE | −6.86 | 4ZAE | −5.27 |
| 26 | 3KL6 | −9.37 | 3M36 | −9.58 | 3KL6 | −11.12 | 3KL6 | −9.39 | 2PHB | −6.74 | 4ZAE | −5.18 |
| 27 | 2W26 | −9.37 | 3KL6 | −9.46 | 2XBX | −11.01 | 2PHB | −9.28 | 2PHB | −6.70 | 4Z0K | −5.02 |
| 28 | 3KL6 | −9.36 | 3KL6 | −9.41 | 2XBX | −11.01 | 3KL6 | −9.28 | 3KL6 | −6.50 | 4ZAE | −4.98 |
| 29 | 3M36 | −9.36 | 2XBX | −9.40 | 3KL6 | −11.01 | 3KL6 | −9.21 | 4ZAE | −6.47 | 4Z0K | −4.94 |
| 30 | 2PHB | −9.35 | 2PHB | −9.38 | 2PHB | −11.00 | 2P16 | −9.11 | 2XBX | −6.44 | 4Z0K | −4.92 |
| 31 | 2P16 | −9.30 | 2XBX | −9.33 | 2XBX | −10.63 | 3KL6 | −9.11 | 2XBX | −6.39 | 3M36 | −4.83 |
| 32 | 3KL6 | −9.30 | 2XBX | −9.28 | 3KL6 | −10.60 | 3KL6 | −9.04 | 2XBX | −6.38 | 4ZAE | −4.83 |
| 33 | 3KL6 | −9.25 | 2W26 | −9.10 | 3KL6 | −10.56 | 3KL6 | −9.01 | 3KL6 | −6.35 | 4ZAE | −4.69 |
| 34 | 2W26 | −9.18 | 3KL6 | −8.89 | 2XBX | −10.30 | 2P16 | −8.92 | 2XBX | −6.25 | 4ZAE | −4.60 |
| 35 | 2W26 | −9.15 | 3KL6 | −8.87 | 3KL6 | −10.25 | 3KL6 | −8.87 | 4ZAE | −6.14 | 3M36 | −4.35 |

| | | | | | | | | | | | | |
|---|---|---|---|---|---|---|---|---|---|---|---|---|
| 36 | 2W26 | −8.92 | 2PHB | −8.85 | 2XBX | −10.22 | 3KL6 | −8.71 | 2XBX | −6.14 | 5TNT | −3.84 |
| 37 | 2PHB | −8.91 | 2W26 | −8.79 | 3KL6 | −9.73 | 2PHB | −8.69 | 2XBX | −6.11 | 5TNT | −3.69 |
| 38 | 3KL6 | −8.90 | 2PHB | −8.78 | 3KL6 | −9.00 | 2PHB | −8.67 | 3KL6 | −5.86 | 4ZAE | −3.51 |
| 39 | 3KL6 | −8.69 | 3KL6 | −8.78 | 5TNT | −8.62 | 2PHB | −8.65 | 3KL6 | −5.71 | 4ZAE | −3.28 |
| 40 | 2PHB | −8.58 | 3KL6 | −8.68 | 5TNT | −8.44 | 3KL6 | −8.46 | 3KL6 | −5.70 | 4ZAE | −3.25 |
| 41 | 2XBX | −8.47 | 2W26 | −8.63 | 3KL6 | −8.41 | 2PHB | −8.42 | 3KL6 | −5.64 | 5TNT | −3.24 |
| 42 | 2XBX | −8.43 | 3KL6 | −8.56 | 5TNT | −8.38 | 2PHB | −8.17 | 3KL6 | −5.51 | 5TNT | −3.20 |
| 43 | 3KL6 | −8.29 | 3KL6 | −8.42 | 5TNO | −7.00 | 3KL6 | −8.13 | 2XBX | −5.38 | 4ZAE | −3.10 |
| 44 | 3KL6 | −8.24 | 3KL6 | −8.39 | 3M36 | −6.37 | 2PHB | −7.98 | 3KL6 | −5.29 | 3M36 | −3.06 |
| 45 | 2XBX | −8.15 | 2W26 | −8.34 | 5TNO | −6.10 | 2PHB | −7.66 | 4ZAE | −5.09 | 5TNT | −2.91 |
| 46 | 2PHB | −8.05 | 2W26 | −8.32 | 5TNO | −6.10 | 5TNT | −7.31 | 3KL6 | −5.07 | 4ZAE | −2.90 |
| 47 | 2XBX | −7.96 | 2W26 | −8.11 | 5TNO | −5.88 | 5EGM | −6.29 | 4ZAE | −5.01 | 4ZAE | −2.89 |
| 48 | 3KL6 | −7.75 | 3KL6 | −7.90 | 5TNO | −5.87 | 5EGM | −6.28 | 3KL6 | −4.99 | 5TNT | −2.87 |
| 49 | 4YZU | −7.71 | 4YZU | −7.45 | 5TNO | −5.41 | 5EGM | −6.12 | 2XBX | −4.98 | 2XBX | −2.80 |
| 50 | 3KL6 | −7.67 | 3M36 | −6.99 | 5EGM | −5.36 | 5TNT | −6.03 | 4Z0K | −4.74 | 2XBX | −2.72 |

**Table S3.** ADMET descriptors for twelve co-crystalized structures using QikProp [4].

| co-crystalized ligand | mol MW | #stars | #amine | #amidine | #acid | #amide | #rotor | #rtvFG | CNS | dipole | SASA | FOSA | FISA | PISA | WPSA | volume-1 | donorHB | accptHB | dip^2/V | ACxDN^.5/SA | glob | QPpolrz | QPlogPC16 | QPlogPoct | QPlogPw | QPlogPo/w | QPlogS | CIQPlogS |
|---|---|---|---|---|---|---|---|---|---|---|---|---|---|---|---|---|---|---|---|---|---|---|---|---|---|---|---|---|
| 5TNT | 499.36 | 1.00 | 0.00 | 0.00 | 0.00 | 0.00 | 3.00 | 0.00 | -2.00 | 5.52 | 759.11 | 258.55 | 193.73 | 216.21 | 90.62 | 1371.03 | 3.00 | 8.50 | 0.02 | 0.02 | 0.79 | 48.85 | 15.27 | 26.20 | 16.47 | 3.32 | -6.79 | -7.07 |
| 5TNO | 423.86 | 1.00 | 0.00 | 0.00 | 0.00 | 0.00 | 3.00 | 0.00 | -2.00 | 7.52 | 681.14 | 122.08 | 223.45 | 302.06 | 33.56 | 1216.06 | 3.00 | 7.50 | 0.05 | 0.02 | 0.81 | 43.47 | 14.15 | 24.16 | 16.06 | 2.46 | -5.56 | -5.97 |
| 5EGM | 550.02 | 2.00 | 0.00 | 0.00 | 0.00 | 0.00 | 2.00 | 0.00 | -2.00 | 12.76 | 820.53 | 156.70 | 206.16 | 439.95 | 17.73 | 1546.71 | 2.00 | 7.50 | 0.11 | 0.01 | 0.79 | 58.66 | 17.79 | 28.95 | 15.97 | 4.86 | -8.08 | -9.18 |
| 4ZAE | 505.41 | 1.00 | 0.00 | 0.00 | 0.00 | 0.00 | 5.00 | 0.00 | -1.00 | 5.83 | 805.83 | 186.10 | 124.74 | 401.22 | 93.78 | 1473.18 | 2.00 | 6.00 | 0.02 | 0.01 | 0.78 | 53.42 | 16.88 | 24.90 | 13.01 | 5.82 | -8.19 | -8.58 |
| 4Z0K | 402.46 | 0.00 | 0.00 | 0.00 | 0.00 | 0.00 | 3.00 | 0.00 | -2.00 | 10.60 | 704.52 | 244.45 | 160.70 | 299.37 | 0.00 | 1260.07 | 2.00 | 6.25 | 0.09 | 0.01 | 0.80 | 45.21 | 13.66 | 22.72 | 12.97 | 3.67 | -6.26 | -6.03 |
| 4YZU | 360.42 | 0.00 | 0.00 | 0.00 | 0.00 | 0.00 | 4.00 | 0.00 | -2.00 | 9.25 | 691.22 | 239.14 | 159.31 | 292.77 | 0.00 | 1197.20 | 2.00 | 6.00 | 0.07 | 0.01 | 0.79 | 41.99 | 13.02 | 21.10 | 12.42 | 3.37 | -5.95 | -5.31 |
| 2W26 | 435.88 | 0.00 | 0.00 | 0.00 | 0.00 | 0.00 | 3.00 | 0.00 | -1.00 | 7.11 | 683.29 | 230.93 | 135.38 | 200.90 | 116.08 | 1220.31 | 1.00 | 10.70 | 0.04 | 0.02 | 0.81 | 42.68 | 12.99 | 22.44 | 15.03 | 2.18 | -4.74 | -4.76 |
| 2P16 | 459.50 | 0.00 | 0.00 | 0.00 | 0.00 | 0.00 | 2.00 | 0.00 | -2.00 | 8.86 | 774.10 | 348.81 | 183.70 | 241.58 | 0.00 | 1399.85 | 2.00 | 10.25 | 0.06 | 0.02 | 0.78 | 50.89 | 14.80 | 26.43 | 17.06 | 2.68 | -6.26 | -5.67 |
| 3KL6 | 479.98 | 0.00 | 0.00 | 0.00 | 0.00 | 1.00 | 5.00 | 0.00 | -2.00 | 9.17 | 769.28 | 304.70 | 192.81 | 199.84 | 71.93 | 1388.39 | 1.00 | 9.70 | 0.06 | 0.01 | 0.78 | 48.11 | 14.74 | 24.00 | 16.13 | 2.44 | -5.21 | -5.22 |
| 2PHB | 484.91 | 0.00 | 0.00 | 0.00 | 0.00 | 1.00 | 4.00 | 0.00 | -1.00 | 10.57 | 784.07 | 178.04 | 111.14 | 401.92 | 92.98 | 1415.70 | 2.00 | 9.20 | 0.08 | 0.02 | 0.78 | 51.77 | 15.95 | 26.76 | 17.60 | 3.62 | -6.26 | -5.83 |
| 3M36 | 532.51 | 0.00 | 1.00 | 0.00 | 0.00 | 0.00 | 6.00 | 0.00 | -1.00 | 6.06 | 820.31 | 112.41 | 161.76 | 391.25 | 154.89 | 1481.11 | 3.00 | 8.50 | 0.02 | 0.02 | 0.77 | 53.00 | 15.78 | 27.96 | 16.95 | 4.12 | -6.38 | -6.83 |
| 2XBX | 532.97 | 2.00 | 0.00 | 0.00 | 0.00 | 0.00 | 5.00 | 0.00 | -2.00 | 15.67 | 843.36 | 137.09 | 187.22 | 415.93 | 103.12 | 1511.11 | 2.00 | 12.50 | 0.16 | 0.02 | 0.76 | 55.08 | 17.58 | 31.18 | 20.03 | 2.98 | -6.58 | -6.12 |

| co-crystalized ligand | QPlogHERG | QPPCaco | QPlogBB | QPPMDCK | QPlogKp | IP(eV) | EA(eV) | #metab | QPlogKhsa | Human OralAbsorption | PercentHuman OralAbsorption | SAfluorine | SAamideO | PSA | #NandO | RuleOfFive | RuleOfThree | #ringatoms | #in34 | #in56 | #noncon | #nonHatm | Jm |
|---|---|---|---|---|---|---|---|---|---|---|---|---|---|---|---|---|---|---|---|---|---|---|---|
| 5TNT | -6.02 | 144.13 | -1.48 | 191.20 | -4.04 | 8.54 | 1.23 | 2.00 | 0.48 | 1.00 | 85.02 | 0.00 | 0.00 | 127.33 | 10.00 | 0.00 | 1.00 | 27.00 | 0.00 | 27.00 | 6.00 | 34.00 | 0.00 |
| 5TNO | -6.07 | 75.33 | -1.81 | 46.16 | -4.29 | 8.27 | 1.32 | 0.00 | 0.29 | 2.00 | 74.93 | 0.00 | 0.00 | 121.48 | 9.00 | 0.00 | 0.00 | 25.00 | 0.00 | 25.00 | 4.00 | 30.00 | 0.00 |
| 5EGM | -7.06 | 109.88 | -1.73 | 56.86 | -3.58 | 8.77 | 1.54 | 3.00 | 1.21 | 1.00 | 78.99 | 0.00 | 0.00 | 124.87 | 10.00 | 1.00 | 1.00 | 35.00 | 0.00 | 35.00 | 4.00 | 40.00 | 0.00 |
| 4ZAE | -7.05 | 650.13 | -0.89 | 1013.77 | -1.93 | 8.93 | 1.39 | 4.00 | 1.16 | 1.00 | 85.43 | 0.00 | 0.00 | 92.60 | 7.00 | 2.00 | 1.00 | 26.00 | 0.00 | 26.00 | 0.00 | 35.00 | 0.00 |
| 4Z0K | -6.18 | 296.47 | -1.29 | 132.93 | -3.14 | 8.47 | 1.07 | 4.00 | 0.65 | 1.00 | 92.66 | 0.00 | 0.00 | 103.23 | 8.00 | 0.00 | 1.00 | 24.00 | 0.00 | 24.00 | 4.00 | 30.00 | 0.00 |
| 4YZU | -6.35 | 305.64 | -1.38 | 137.38 | -3.04 | 9.01 | 1.19 | 3.00 | 0.48 | 3.00 | 91.15 | 0.00 | 0.00 | 98.12 | 7.00 | 0.00 | 1.00 | 20.00 | 0.00 | 20.00 | 0.00 | 27.00 | 0.00 |
| 2W26 | -5.59 | 515.36 | -0.71 | 1044.82 | -3.02 | 9.05 | 1.41 | 3.00 | -0.34 | 3.00 | 88.26 | 0.00 | 0.00 | 116.92 | 8.00 | 0.00 | 0.00 | 22.00 | 0.00 | 22.00 | 5.00 | 29.00 | 0.01 |
| 2P16 | -6.22 | 179.40 | -1.56 | 77.24 | -3.86 | 9.14 | 0.46 | 4.00 | 0.30 | 1.00 | 82.97 | 0.00 | 0.00 | 133.62 | 9.00 | 0.00 | 1.00 | 27.00 | 0.00 | 27.00 | 6.00 | 34.00 | 0.00 |
| 3KL6 | -4.63 | 90.84 | -1.66 | 154.36 | -3.89 | 9.22 | 1.38 | 1.00 | -0.12 | 3.00 | 76.26 | 0.00 | 26.28 | 125.04 | 8.00 | 0.00 | 0.00 | 22.00 | 0.00 | 22.00 | 8.00 | 32.00 | 0.00 |
| 2PHB | -5.67 | 624.65 | -0.68 | 1383.29 | -1.77 | 8.84 | 0.61 | 2.00 | 0.13 | 1.00 | 100.00 | 21.44 | 18.38 | 103.98 | 8.00 | 0.00 | 1.00 | 23.00 | 0.00 | 23.00 | 4.00 | 34.00 | 0.00 |
| 3M36 | -7.91 | 72.24 | -0.86 | 225.53 | -4.61 | 9.46 | 0.85 | 3.00 | 0.61 | 1.00 | 71.36 | 154.89 | 0.00 | 110.70 | 7.00 | 1.00 | 1.00 | 23.00 | 0.00 | 23.00 | 0.00 | 37.00 | 0.00 |
| 2XBX | -7.48 | 166.16 | -1.67 | 261.07 | -3.03 | 9.03 | 0.90 | 3.00 | -0.01 | 1.00 | 71.18 | 31.56 | 0.00 | 139.13 | 9.00 | 1.00 | 1.00 | 23.00 | 0.00 | 23.00 | 4.00 | 36.00 | 0.00 |

| | |
|---|---|
| (white) | Within range |
| (red) | Out of range |

.

| Property or Descriptor | Description | Range or recommended values |
|---|---|---|
| **molecule name** | Molecule name taken from the title line in the input structure file. If the title line is blank, the input file name is used. | - |
| **#stars** | Number of property or descriptor values that fall outside the 95% range of similar values for known drugs. Outlying descriptors and predicted properties are denoted with asterisks (*) in the .out file. A large number of stars suggests that a molecule is less drug-like than molecules with few stars. The following properties and descriptors are included in the determination of #stars: MW, dipole, IP, EA, SASA, FOSA, FISA, PISA, WPSA, PSA, volume, #rotor, donorHB, accptHB, glob, QPpolrz, | 0–5 |

| | | |
|---|---|---|
| | QPlogPC16, QPlogPoct, QPlogPw, QPlogPo/w, logS, QPLogKhsa, QPlogBB, #metabol | |
| **#amine** | Number of non-conjugated amine groups. | 0–1 |
| **#amidine** | Number of amidine and guanidine groups. | 0 |
| **#acid** | Number of carboxylic acid groups. | 0–1 |
| **#amide** | Number of non-conjugated amide groups. | 0–1 |
| **#rotor** | Number of non-trivial (not CX3), non-hindered (not alkene, amide, small ring) rotatable bonds. | 0–15 |
| **#rtvFG** | Number of reactive functional groups; the specific groups are listed in the *jobname* .out file. The presence of these groups can lead to false positives in HTS assays and to decomposition, reactivity, or toxicity problems *in vivo*.. | 0–2 |
| **CNS** | Predicted central nervous system activity on a −2 (inactive) to +2 (active) scale. | −2 (inactive), +2 (active) |
| **mol_MW** | Molecular weight of the molecule. | 130.0–725.0 |
| **dipole†** | Computed dipole moment of the molecule. | 1.0–12.5 |
| **SASA** | Total solvent accessible surface area (SASA) in square angstroms using a probe with a 1.4 Å radius. | 300.0–1000.0 |
| **FOSA** | Hydrophobic component of the SASA (saturated carbon and attached hydrogen). | 0.0–750.0 |
| **FISA** | Hydrophilic component of the SASA (SASA on N, O, H on heteroatoms, carbonyl C). | 7.0–330.0 |
| **PISA** | π (carbon and attached hydrogen) component of the SASA. | 0.0–450.0 |
| **WPSA** | Weakly polar component of the SASA (halogens, P, and S). | 0.0–175.0 |
| **volume** | Total solvent-accessible volume in cubic angstroms using a probe with a 1.4 Å radius. | 500.0–2000.0 |
| **donorHB** | Estimated number of hydrogen bonds that would be donated by the solute to water molecules in an aqueous solution. Values are averages taken over a number of configurations, so they can be non-integer. | 0.0–6.0 |
| **accptHB** | Estimated number of hydrogen bonds that would be accepted by the solute from water molecules in an aqueous solution. Values are averages taken over a number of configurations, so they can be non-integer. | 2.0–20.0 |
| **dip^2/V†** | Square of the dipole moment divided by the molecular volume. This is the key term in the Kirkwood-Onsager equation for the free energy of solvation of a dipole with volume V. | 0.0–0.13 |
| **ACxDN^.5/SA** | Index of cohesive interaction in solids. | 0.0–0.05 |
| **glob** | Globularity descriptor. Globularity is 1.0 for a spherical molecule. | 0.75–0.95 |
| **QPpolrz** | Predicted polarizability in cubic angstroms. | 13.0–70.0 |
| **QPlogPC16** | Predicted hexadecane/gas partition coefficient. | 4.0–18.0 |
| **QPlogPoct‡** | Predicted octanol/gas partition coefficient. | 8.0–35.0 |

| | | |
|---|---|---|
| **QPlogPw** | Predicted water/gas partition coefficient. | 4.0–45.0 |
| **QPlogPo/w** | Predicted octanol/water partition coefficient. | −2.0–6.5 |
| **QPlogS** | Predicted aqueous solubility, log S. S in mol $dm^{-3}$ is the concentration of the solute in a saturated solution that is in equilibrium with the crystalline solid. | −6.5–0.5 |
| **CIQPlogS** | Conformation-independent predicted aqueous solubility, log S. S in mol $dm^{-3}$ is the concentration of the solute in a saturated solution that is in equilibrium with the crystalline solid. | −6.5–0.5 |
| **QPlogHERG** | Predicted $IC_{50}$ value for blockage of HERG $K^+$ channels. | concern below −5 |
| **QPPCaco** | Predicted apparent Caco-2 cell permeability in nm/sec. Caco-2 cells are a model for the gut-blood barrier. QikProp predictions are for non-active transport. | < 25 poor, > 500 great |
| **QPlogBB** | Predicted brain/blood partition coefficient. Note: QikProp predictions are for orally delivered drugs so, for example, dopamine and serotonin are CNS negative because they are too polar to cross the blood-brain barrier | −3.0–1.2 |
| **QPPMDCK** | Predicted apparent MDCK cell permeability in nm/sec. MDCK cells are considered to be a good mimic for the blood-brain barrier. QikProp predictions are for non-active transport. | < 25 poor, > 500 great |
| **QPlogKp** | Predicted skin permeability, log $K_p$. | −8.0–−1.0 |
| **IP(ev)†** | PM3 calculated ionization potential (negative of HOMO energy). | 7.9–10.5 |
| **EA(eV)†** | PM3 calculated electron affinity (negative of LUMO energy). | −0.9–1.7 |
| **#metab‡** | Number of likely metabolic reactions. | 1–8 |
| **QPlogKhsa** | Prediction of binding to human serum albumin. | −1.5–1.5 |
| **HumanOralAbsorption** | Predicted qualitative human oral absorption: 1, 2, or 3 for low, medium, or high. The text version is reported in the output. The assessment uses a knowledge-based set of rules, including checking for suitable values of PercentHumanOralAbsorption, number of metabolites, number of rotatable bonds, logP, solubility and cell permeability. | - |
| **PercentHumanOralAbsorption** | Predicted human oral absorption on 0 to 100% scale. The prediction is based on a quantitative multiple linear regression model. This property usually correlates well with HumanOralAbsorption, as both measure the same property. | > 80% is high < 25% is poor |
| **SAFluorine** | Solvent-accessible surface area of fluorine atoms. | 0.0–100.0 |
| **SAamideO** | Solvent-accessible surface area of amide oxygen atoms. | 0.0–35.0 |
| **PSA** | Van der Waals surface area of polar nitrogen and oxygen atoms and carbonyl carbon atoms. | 7.0–200.0 |
| **#NandO** | Number of nitrogen and oxygen atoms. | 2–15 |
| **RuleOfFive** | Number of violations of Lipinski's rule of five. The rules are: mol_MW < 500, QPlogPo/w < 5, donorHB ≤ 5, accptHB ≤ 10. Compounds that satisfy these rules are considered drug-like. (The "five" refers to the limits, which are multiples of 5.) | maximum is 4 |

| | | |
|---|---|---|
| **RuleOfThree** | Number of violations of Jorgensen's rule of three. The three rules are: QPlogS > -5.7, QP PCaco > 22 nm/s, # Primary Metabolites < 7. Compounds with fewer (and preferably no) violations of these rules are more likely to be orally available. | maximum is 3 |
| **#ringatoms** | Number of atoms in a ring | - |
| **#in34** | Number of atoms in 3- or 4-membered rings | - |
| **#in56** | Number of atoms in 5- or 6-membered rings | - |
| **#noncon** | number of ring atoms not able to form conjugated aromatic systems (e.g. $sp^3$ C). | - |
| **#nonHatm** | Number of heavy atoms (nonhydrogen atoms) | - |
| **Jm** | Predicted maximum transdermal transport rate, $K_p \times MW \times S$ (μ g $cm^{-2}\ hr^{-1}$). $K_p$ and S are obtained from the aqueous solubility and skin permeability, QPlogKp and QPlogS. This property is only written to the output file: it is not used in any other calculations. | - |

## References

1.Abraham, M.J.; Murtola, T.; Schulz, R.; Páll, S.; Smith, J.C.; Hess, B.; Lindahl, E. GROMACS: High Performance Molecular Simulations through Multi-Level Parallelism from Laptops to Supercomputers. SoftwareX 2015, 1–2, 19–25.
2.Lindorff-Larsen, K.; Piana, S.; Palmo, K.; Maragakis, P.; Klepeis, J. L.; Dror, R. O.; Shaw, D. E.. Improved side-chain torsion potentials for the Amber ff99SB protein force field. Proteins, 2010, 78, 1950–1958.
3.Wang, J.; Wang, W.; Kollman, P.A.; Case, D.A. Automatic Atom Type and Bond Type Perception in Molecular Mechanical Calculations. J. Mol. Graph. Model 2006, 25, 247–260.
4. Schrödinger Release 2021-3: QikProp, Schrödinger, LLC, New York, NY, 2021.
